# Emerging Trends in Patients Hospitalised With Cirrhosis—Aetiologies, Complications and Outcomes Compared to Other Chronic Health Conditions

**DOI:** 10.1111/apt.70287

**Published:** 2025-07-28

**Authors:** Julian Pohl, Nirbaanjot Walia, Lukas Hofmann, Lena Wolters, Martin Schulz, Matthias Reinhardt, Brigitta Globke, Frank Tacke, Thomas Berg, Cornelius Engelmann

**Affiliations:** ^1^ Department of Hepatology and Gastroenterology Charité—Universitätsmedizin Berlin, Campus Virchow‐Klinikum (CVK) and Campus Charité Mitte (CCM) Berlin Germany; ^2^ Division of Hepatology, Department of Medicine II Leipzig University Medical Center Leipzig Germany; ^3^ Department of Medicine B Muenster University Hospital Muenster Germany; ^4^ Department of Surgery Campus Charité Mitte and Campus Virchow‐Klinikum, Charité‐Universitätsmedizin Berlin Germany; ^5^ Institute for Liver and Digestive Health, Royal Free Campus University College London London UK

**Keywords:** alcohol related liver disease, epidemiology, liver cirrhosis, public health

## Abstract

**Background:**

The aetiologies, complications and overall burden of cirrhosis have undergone changes in recent years.

**Aims:**

This study aimed to assess the emerging trends in cirrhosis and its complications whilst also comparing them to other major chronic health conditions.

**Methods:**

This retrospective study covering 2011–2022 was conducted at the Charité University Hospital Berlin and University Hospital Leipzig (validation cohort), Germany. ICD‐10 codes were used to identify patients with cirrhosis, its complications and comorbidities. Changes in demographics, aetiologies, complication rates and inpatient mortality were assessed. Trends were compared against major chronic health conditions.

**Results:**

24,567 (Berlin) and 14,141 (Leipzig) inpatient admissions for cirrhosis were identified. Overall admission numbers have been decreasing; however, in‐hospital mortality and complication rates have been increasing. Alcohol‐related liver disease was persistently the most common cause of cirrhosis (42%). Mortality rates for admissions with variceal haemorrhage and gastrointestinal bleeding have increased from 15.53% and 10.53% in 2011 to 27.69% and 31.25% in 2022, respectively. Cirrhosis admissions had some of the highest rates of inpatient mortality (7.45%), despite being considerably younger and less comorbid than admissions with other chronic health conditions. Over time, the median age of admissions with other chronic diseases increased considerably more than admissions with cirrhosis. In the validation cohort, high inpatient mortality, increasing GI bleeding associated mortality, and complications rates were confirmed.

**Conclusion:**

Cirrhosis remains a significant clinical and public health challenge and is falling behind other major comorbidities with regard to inpatient outcomes. A focus on improved resource allocation and management options is warranted.

## Graphical abstract

Rates of inpatient mortality and complication rates in cirrhosis remain high. With regard to inpatient outcomes, cirrhosis is falling behind other chronic health conditions.

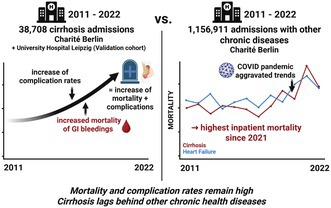

## 1 Introduction

As a critical endpoint of liver disease, cirrhosis represents a significant clinical and public health challenge, as it continues to pose high rates of morbidity and mortality worldwide [1, 2, 3, 4]. In Europe, the prevalence, causes and complications of liver disease are undergoing changes secondary to healthcare interventions, lifestyle changes and demographic shifts [1, 5]. Effective viral hepatitis prevention and management strategies have considerably reduced the prevalence and burden of the hepatitis B (HBV) and C (HCV) virus infections.

In contrast, rising rates of factors contributing to metabolic syndrome are concerning for an increasing incidence of metabolic dysfunction‐associated steatotic liver disease (MASLD) [6, 7, 8]. Although alcohol‐related liver disease (ALD) persists as the major cause of cirrhosis in the Western world, [8, 9] shifting patterns in alcohol consumption have been noted [10, 11]. Changing demographics such as aging populations and immigration are similarly likely to influence health outcomes in patients with cirrhosis [12, 13]. Similarly, the impact of the COVID‐19 pandemic warrants consideration. The recurrent surges in COVID‐19 cases posed significant challenges to healthcare delivery and diverted attention from non‐COVID‐related conditions. How these developments have impacted the landscape of liver disease requires further evaluation [14].

This study therefore aimed to assess the changing trends in the aetiologies, complications and burdens of cirrhosis, using a real‐world data set from a large healthcare centre in Berlin, Germany. Furthermore, the study sought to compare the burden of cirrhosis against patients with other major chronic health conditions, such as chronic obstructive pulmonary disease (COPD), chronic kidney disease (CKD), congestive cardiac failure (CCF), ischaemic heart disease (IHD), diabetes and malignancy, to assess the distinct challenges cirrhosis poses to patients and healthcare systems.

## 2 Methods

### 2.1 Study Design, Setting and Population

This was a retrospective observational study reviewing hospital admissions to the Charité—Universitätsmedizin Berlin, Germany, from 2011 to 2022. Admissions with a diagnosis of cirrhosis, either as a comorbidity or as a primary reason for admission, were included. Hospital admissions with other major chronic health conditions, such as COPD, CKD, CCF, IHD, diabetes and malignancy were also included for comparison against admissions with cirrhosis. A post hoc validation cohort at University Hospital Leipzig, Germany, was obtained to evaluate key trends from 2011 to 2022.

### 2.2 Data Collection

ICD‐10‐GM codes (K74 and K70.3) were used to extract patient admission episodes with a diagnosis of cirrhosis and were used to identify complications of cirrhosis present during the admission. These codes were similarly used to identify aetiologies of cirrhosis, such as ALD, MASLD (using codes for non‐alcoholic fatty liver disease and non‐alcoholic steatohepatitis), HBV, HCV and others. Patients with primary sclerosing cholangitis (PSC) could not accurately be identified, given that the diagnosis only received a specific ICD‐10‐GM code in 2022. ICD‐10‐GM codes were also used to identify patients with other chronic health conditions and comorbidities (Supporting Information S1). Available data included patient age, sex, date of admission, duration of admission, in‐hospital mortality and recorded complications and comorbidities. The study was approved by the institutional ethics committee of the Charité—Universitätsmedizin Berlin (ethical approval number EA2/091/19) and was carried out in accordance with the Declaration of Helsinki.

### 2.3 Statistical Analysis

Statistical analyses were conducted using R software version 4.3.2. Descriptive statistics were generated, with median and interquartile range (IQR) provided for variables not normally distributed and mean and standard deviation (SD) for those normally distributed. Figures on the yearly population of Berlin were obtained from the United Nations World Population Prospects 2022 [15] and nationwide COVID‐19 data was obtained from OurWorldinData [16]. Age‐adjusted mortality rates were calculated using the European standard and compared with the chronic health conditions assessed [17].

Ad‐hoc analyses included quantile regression to evaluate changes in the age of patients with specified health conditions over time. Spearman's correlation was used to assess relationships between nationwide COVID‐19 hospitalisations and deaths with cirrhosis admissions and mortality rates. The median monthly admissions and mortality rates were compared between the pre‐COVID‐19 and COVID‐19 periods (defined as March 2020 to December 2022) using the Wilcoxon rank‐sum test.

## 3 Results

### 3.1 Demographic Data

A total of 24,567 inpatient hospital admissions with cirrhosis were identified from 2011 to 2022, with a median age of 62 (IQR 53–70). 34.68% of admissions were female. The yearly trends are described in Table 1. The number of admissions per year has been decreasing in recent years, from a peak of 2348 in 2014, down to 1684 in 2022, with a similar trend being seen when adjusted for the city's population. The median age of cirrhosis admissions displays a slight upward trend with fluctuations from 60.42 years in 2011 to 63.17 years in 2017 and has remained stable at 62 years in recent years. Yearly median MELD and MELD‐Na scores ranged from 11.8 to 14.0 and 12.7 to 15.6, respectively, without a clear trend.

**TABLE 1:** Characteristics and complications of patients admitted with cirrhosis.

Year	2011	2012	2013	2014	2015	2016	2017	2018	2019	2020	2021	2022	Total
Admissions	2146	2026	2083	2348	2269	2248	2318	1965	1924	1974	1582	1684	24,567
Admissions per 100,000 population	61.97	58.29	59.72	67.07	64.57	63.75	65.50	55.32	54.09	55.42	44.35	47.16	—
In‐hospital mortality (*n*)	150	148	135	168	160	154	145	140	163	153	167	148	1831
In‐hospital mortality rate	0.07	0.07	0.06	0.07	0.07	0.07	0.06	0.07	0.08	0.08	0.11	0.09	0.07
Age (median years, IQR)	60 (54–69)	62 (53–70)	62 (53–70)	61 (53–71)	62 (54–71)	63 (53–72)	63 (54–72)	63 (53–70)	62 (53–69)	62 (55–69)	62 (53–70)	62 (54–70)	62 (53–70)
Days of admission (mean (SD))	12.27 (19.76)	12.53 (19.11)	11.55 (18.51)	11.69 (17.29)	11.16 (17.73)	10.93 (16.53)	10.01 (15.82)	10.80 (17.05)	10.63 (18.15)	10.60 (15.36)	11.18 (15.66)	12.39 (17.53)	11.29 (17.46)
Sex (% female)	0.33	0.33	0.34	0.36	0.35	0.36	0.35	0.32	0.34	0.37	0.39	0.32	0.35
Liver transplants (*n*)	90	70	62	56	70	64	54	45	38	49	39	51	688
ICU admissions	500	504	486	546	513	503	469	388	444	500	434	490	5777
	0.23	0.25	0.23	0.23	0.23	0.22	0.20	0.20	0.23	0.25	0.27	0.29	0.24
TIPS	83	77	66	62	45	49	28	40	50	37	55	48	640
	0.04	0.04	0.03	0.03	0.02	0.02	0.01	0.02	0.02	0.03	0.03	0.03	0.03
MELD at admission (median, IQR)	11.8 (8.8–15.9)	12.4 (9.4–17.1)	13.1 (9.5–18.6)	13.0 (9.6–18.2)	13.4 (9.5–18.7)	12.3 (9.1–17.9)	12.3 (9.0–18.2)	11.7 (8.8–17.9)	13.9 (9.9–19.3)	14.0 (10.0–19.6)	13.0 (9.1–20.2)	12.9 (8.9–19.1)	12.9 (9.4–18.6)
MELD‐Na at admission	14.0	14.5	14.9	14.4	14.7	13.6	13.2	12.7	15.1	15.6	14.7	14.5	14.4
(median, IQR)	(9.8–19.3)	(10.4–20.7)	(10.5–23.3)	(10.0–22.5)	(10.1–22.3)	(9.5–21.0)	(9.4–20.7)	(9.1–20.5)	(10.4–22.2)	(10.7–22.5)	(9.7–23.7)	(9.7–22.0)	(9.9–21.9)
*Aetiology (n, proportion of total admissions)*													
Alcohol‐related liver disease	982	880	862	940	836	929	926	829	854	866	712	742	10,358
	0.46	0.43	0.41	0.40	0.37	0.41	0.40	0.42	0.44	0.44	0.45	0.44	0.42
MASLD	57	42	38	80	80	89	92	65	53	66	64	60	786
	0.03	0.02	0.02	0.03	0.04	0.04	0.04	0.03	0.03	0.03	0.04	0.04	0.03
Hepatitis C	302	216	39	43	41	47	14	19	22	32	24	38	837
	0.14	0.11	0.02	0.02	0.02	0.02	0.01	0.01	0.01	0.02	0.02	0.02	0.03
Hepatitis B	103	72	18	26	20	36	52	55	56	52	68	56	614
	0.05	0.04	0.01	0.01	0.01	0.02	0.02	0.03	0.03	0.03	0.04	0.03	0.02
Primary biliary cholangitis	68	64	101	89	58	83	83	67	95	90	83	59	940
	0.03	0.03	0.05	0.04	0.03	0.04	0.04	0.03	0.05	0.05	0.05	0.04	0.04
Autoimmune hepatitis	36	46	46	73	39	57	74	59	72	57	39	42	640
	0.02	0.02	0.02	0.03	0.02	0.03	0.03	0.03	0.04	0.03	0.02	0.02	0.03
Haemachromatosis	28	17	25	21	18	17	12	6	17	10	5	4	180
	0.01	0.01	0.01	0.01	0.01	0.01	0.01	0.00	0.01	0.01	0.00	0.00	0.01
Congestive hepatopathy	0	3	3	11	12	15	20	8	39	35	7	16	169
	0.00	0.00	0.00	0.00	0.01	0.01	0.01	0.00	0.02	0.02	0.00	0.01	0.01
Toxic	10	7	12	19	12	23	9	6	16	15	14	10	153
	0.00	0.00	0.01	0.01	0.01	0.01	0.00	0.00	0.01	0.01	0.01	0.01	0.01
Wilsons	5	10	8	10	11	14	13	13	11	9	8	8	120
	0.00	0.00	0.00	0.00	0.00	0.01	0.01	0.01	0.01	0.00	0.01	0.00	0.00
BCS	4	10	14	11	11	11	7	2	2	9	6	6	93
	0.00	0.00	0.01	0.00	0.00	0.00	0.00	0.00	0.00	0.00	0.00	0.00	0.00
Undefined/other	679	743	972	1084	1170	1014	1079	881	741	784	605	704	10,456
	0.32	0.37	0.47	0.46	0.52	0.45	0.47	0.45	0.39	0.40	0.38	0.42	0.43
*Complications (n, proportion of total admissions)*													
Ascites	521	505	526	592	670	565	567	549	672	594	480	501	6742
	0.24	0.25	0.25	0.25	0.30	0.25	0.24	0.28	0.35	0.30	0.30	0.30	0.27
Hepatic encephalopathy	371	301	344	394	429	406	357	351	392	393	286	308	4332
	0.17	0.15	0.17	0.17	0.19	0.18	0.15	0.18	0.20	0.20	0.18	0.18	0.18
Variceal haemorrhage	103	85	95	118	79	89	72	70	92	86	79	65	1033
	0.05	0.04	0.05	0.05	0.03	0.04	0.03	0.04	0.05	0.04	0.05	0.04	0.04
Other gastrointestinal bleeding	76	44	80	65	90	102	70	44	49	61	64	64	809
	0.04	0.02	0.04	0.03	0.04	0.05	0.03	0.02	0.03	0.03	0.04	0.04	0.03
Hepatorenal syndrome	114	123	130	133	168	146	141	135	171	93	97	89	1540
	0.05	0.06	0.06	0.06	0.07	0.06	0.06	0.07	0.09	0.05	0.06	0.05	0.06
Bacterial peritonitis	117	123	107	125	118	120	106	94	124	117	121	125	1397
	0.05	0.06	0.05	0.05	0.05	0.05	0.05	0.05	0.06	0.06	0.08	0.07	0.06
Other bacterial infections	486	480	453	538	578	674	675	532	538	612	526	534	6626
	0.23	0.24	0.22	0.23	0.25	0.30	0.29	0.27	0.28	0.31	0.33	0.32	0.27
Hepatocellular carcinoma	416	365	403	454	448	417	496	437	385	373	304	384	4882
	0.19	0.18	0.19	0.19	0.20	0.19	0.21	0.22	0.20	0.19	0.19	0.23	0.20
Portal vein thrombosis	113	98	87	101	118	110	96	91	84	85	70	92	1145
	0.05	0.05	0.04	0.04	0.05	0.05	0.04	0.05	0.04	0.04	0.04	0.05	0.05
Sarcopenia	NA	NA	NA	NA	11	17	68	52	48	47	69	83	395
	NA	NA	NA	NA	0.00	0.01	0.03	0.03	0.02	0.02	0.04	0.05	0.02
Malnutrition	121	119	122	129	139	215	165	138	132	170	132	152	1734
	0.06	0.06	0.06	0.05	0.06	0.10	0.07	0.07	0.07	0.09	0.08	0.09	0.07
Osteoporosis	74	76	84	93	108	112	80	63	43	29	35	18	815
	0.03	0.04	0.04	0.04	0.05	0.05	0.03	0.03	0.02	0.01	0.02	0.01	0.03
*Proportion of cirrhosis admissions with various comorbidities*													
Diabetes	0.39	0.39	0.40	0.38	0.37	0.37	0.36	0.38	0.31	0.30	0.30	0.30	0.36
Obesity	0.08	0.08	0.08	0.08	0.10	0.12	0.12	0.08	0.06	0.04	0.05	0.06	0.08
Ischaemic heart disease	0.09	0.11	0.13	0.11	0.12	0.13	0.15	0.12	0.08	0.08	0.06	0.07	0.11
Congestive cardiac failure	0.08	0.09	0.09	0.10	0.11	0.13	0.13	0.09	0.08	0.09	0.08	0.09	0.09
Chronic obstructive pulmonary disease	0.08	0.08	0.09	0.10	0.10	0.10	0.08	0.05	0.04	0.06	0.05	0.05	0.07
Chronic kidney disease	0.14	0.17	0.18	0.18	0.20	0.24	0.22	0.18	0.13	0.15	0.13	0.12	0.17
Malignancy	0.29	0.28	0.29	0.29	0.31	0.30	0.33	0.32	0.28	0.29	0.28	0.33	0.30
Cerebrovascular disease	0.02	0.02	0.02	0.02	0.02	0.03	0.03	0.02	0.02	0.02	0.03	0.03	0.10

Abbreviations: BCS = Budd‐Chiari syndrome, IQR = interquartile range, MASLD = metabolic dysfunction‐associated steatotic liver disease, MELD = model for end‐stage liver disease, SD = standard deviation.

The most common comorbidity was diabetes, affecting 36.25% of cirrhosis admissions, followed by malignancy at 29.83%. The least common comorbidities included obesity (8.84%) and COPD (7.43%). As can be seen in Table 1 and Figure S1, the proportion of patients admitted with comorbidities such as diabetes, IHD, COPD and obesity reached a peak during the middle of the study period but has since steadily declined.

### 3.2 Duration of Hospital Admission, Complications of Cirrhosis and Inpatient Mortality

The average duration of admission was 11.29 days (SD 13.30) and has fluctuated but remained relatively stable over time (Table 1). An overall in‐hospital mortality rate of 7.45% was observed. The yearly inpatient mortality rate has trended upwards, but the overall numbers of inpatient deaths remain stable, ranging from 135 to 167 deaths per year. A total of 688 liver transplants occurred over the observation period and have generally declined from a peak of 90 in 2011 to a low of 38 in 2019. A median of 67 transplants occurred a year from 2011 to 2016, compared to just 47 per year from 2017 to 2022. The number of patients who underwent transjugular intrahepatic portosystemic shunt (TIPS) implantation was the highest in 2011 with a total of 83 and declined to 28 in 2017. Since then, TIPS numbers have increased to approximately 50 per year.

The relative number of patients with at least one intensive‐care unit (ICU) admission within their hospitalisation was stable from 2011 to 2015 at around 23%, then declined to 20% in 2018 and since 2019 trended upwards to a maximum of 29% in 2022.

**FIGURE 1:**
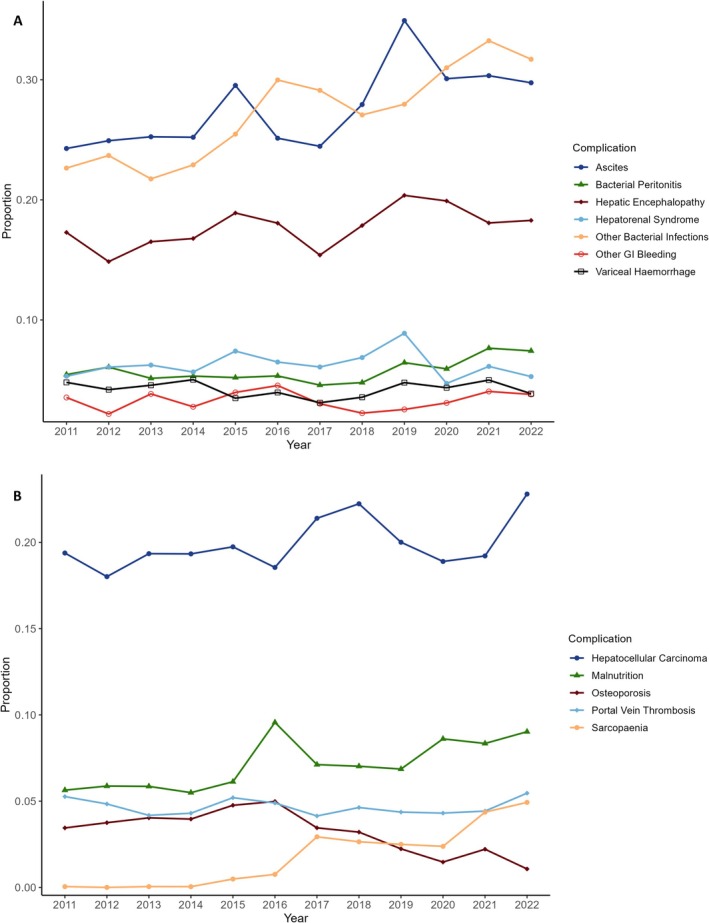
Proportion of cirrhosis admissions with various complications over time: (A) Ascites, bacterial peritonitis, other bacterial infections, hepatic encephalopathy, hepatorenal syndrome, variceal haemorrhage, other gastrointestinal bleeding. (B) Hepatocellular carcinoma, malnutrition, osteoporosis, portal vein thrombosis, sarcopenia.

Ascites was the most common complication of cirrhosis, affecting 27.44% of all admissions, followed by bacterial infections other than bacterial peritonitis (26.97%) and hepatic encephalopathy (HE, 17.63%). Over time, there has been fluctuating but generally rising proportions of admissions with ascites, HE, bacterial peritonitis, other bacterial infections, hepatocellular carcinoma (HCC), malnutrition and sarcopenia (Figure 1).

The overall number of most cirrhosis complications, however, peaked during the middle of the observation period and has been decreasing since. The exceptions to this include bacterial peritonitis, which has had relatively consistent numbers and sarcopenia, which has been increasing (Table 1).

As expected, patients with hepatorenal syndrome (HRS) and bacterial peritonitis had the highest likelihood of inpatient mortality (33.44% and 33.21%) respectively (Table S2). Pneumonia was more common than bacterial peritonitis (8.19% vs. 5.67%) and was associated with a higher inpatient mortality (37.25% vs. 33.21%, Figure S2). Over time, the mortality rate with variceal haemorrhage and other gastrointestinal bleeding (GIB) has been fluctuating but generally increasing, with 15.53% and 10.53% in 2011 to 27.69% and 31.25% in 2022 respectively. These findings can be correlated with a yearly increase of MELD scores in patients with variceal haemorrhage as well as other gastrointestinal haemorrhage (Table S6, Figure S13A–C). Mortality rates for the other complications have fluctuated over the years without a clear trend.

### 3.3 Aetiology of Cirrhosis

**FIGURE 2:**
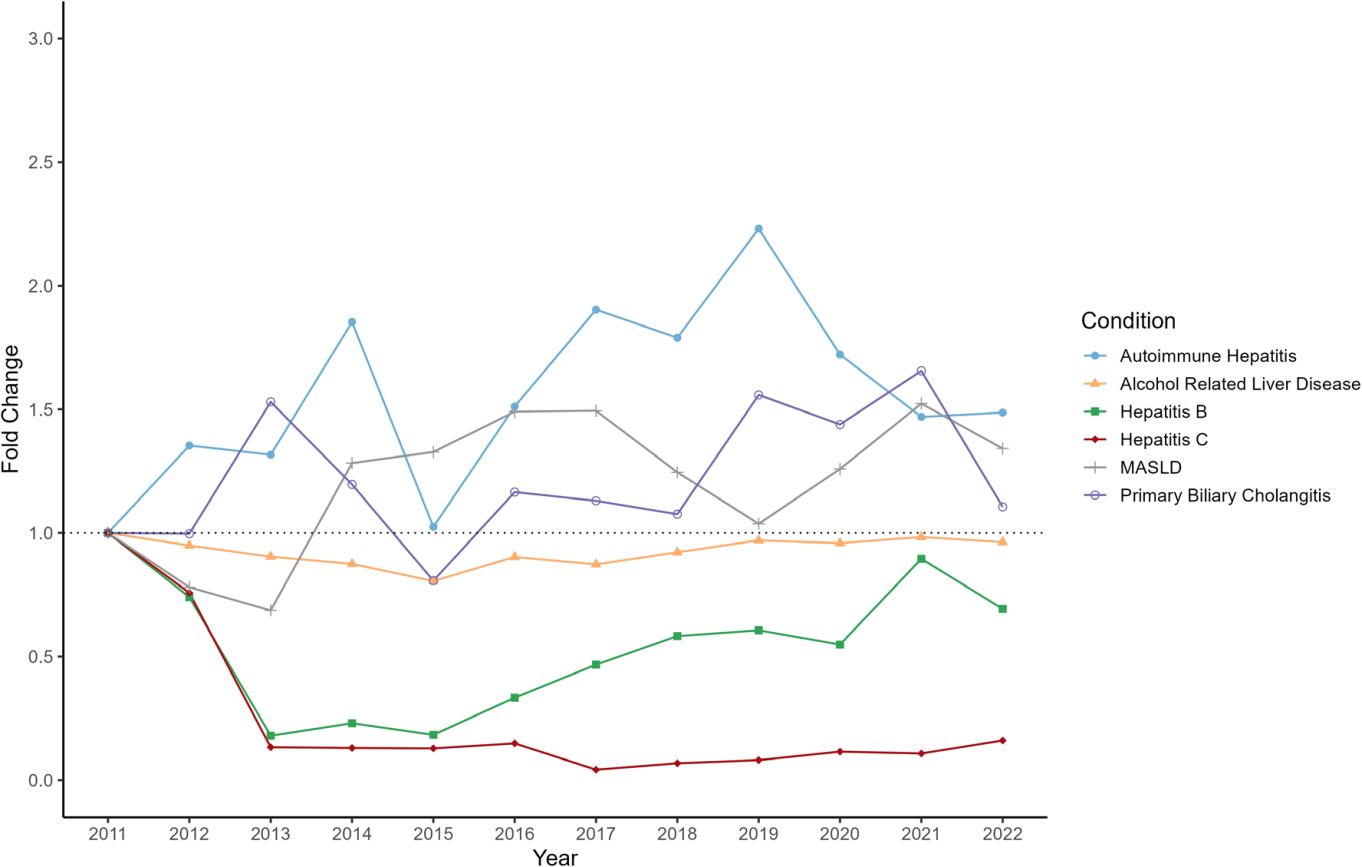
Fold‐change in the proportion of cirrhosis admissions with particular aetiologies.

As described in Table 1, ALD was overwhelmingly the most common listed aetiology of cirrhosis, making up 42.16% of total admissions, followed by Primary Biliary Cholangitis (PBC, 3.82%), HCV (3.41%) and MASLD (3.20%). Over the study period, the proportion of cirrhosis admissions with ALD remained consistent (Figure 2). HCV and HBV as aetiologies significantly reduced from 2011 to 2013, with admissions for HBV steadily increasing since. The proportion of cirrhosis admissions with MASLD generally rose from 2.66% in 2011 to 3.97% by 2017, but has since fluctuated without a clear rise. Similarly, the gross number of MASLD peaked at 92 in 2017 but has since decreased to 60 by 2022.

### 3.4 Demographics by Aetiology

Patients with congestive hepatopathy displayed the highest age (median 76 years, (IQR 62–79)), whilst patients with Wilson Disease (WD) and Budd‐Chiari Syndrome (BCS) displayed the youngest (median age 42 years, (IQR 32–54) and 46 years (IQR 32–58), respectively) (Table 2). There were no clear yearly trends in the age at admission of the various aetiologies over the study period.

**TABLE 2:** Demographics, complications and inpatient mortality of cirrhosis admissions by aetiology.

Aetiology	ALD	MASLD	HCV	HBV	PBC	AIH	HCH	Congestive	Toxic	Wilsons	BCS
Age (median, IQR)	61	62	59	59	61	54	63	76	58	58	46
	54–68	53–69	52–67	52–65	52–71	40–63	56–71	62–79	46–68	46–68	32–58
Proportion female	0.26	0.40	0.36	0.18	0.82	0.68	0.29	0.18	0.42	0.57	0.46
Days of admission (mean, (SD))	11.63	13.50	14.34	12.12	9.49	11.72	15.82	13.78	25.81	12.40	22.78
	(17.17)	(20.82)	(20.61)	(16.97)	(15.90)	(17.87)	(26.05)	(20.62)	(33.13)	(16.26)	(36.89)
Inpatient mortality rate	0.09	0.08	0.09	0.07	0.03	0.07	0.10	0.09	0.13	0.08	0.12
Proportion of admissions with cirrhosis with specific complications											
Ascites	0.32	0.21	0.29	0.29	0.15	0.27	0.36	0.71	0.36	0.28	0.46
Hepatic encephalopathy	0.22	0.13	0.20	0.20	0.07	0.18	0.19	0.08	0.30	0.37	0.15
Variceal haemorrhage	0.06	0.04	0.06	0.04	0.03	0.03	0.04	0.02	0.07	0.05	0.08
Other gastrointestinal bleeding	0.04	0.05	0.04	0.02	0.02	0.01	0.03	0.04	0.05	0.03	0.00
Hepatorenal syndrome	0.08	0.05	0.06	0.06	0.03	0.05	0.11	0.07	0.10	0.08	0.13
Bacterial peritonitis	0.06	0.06	0.08	0.06	0.03	0.06	0.09	0.07	0.09	0.07	0.16
Other bacterial infections	0.29	0.28	0.29	0.23	0.20	0.24	0.26	0.27	0.40	0.24	0.37
Hepatocellular carcinoma	0.18	0.12	0.28	0.46	0.04	0.04	0.27	0.02	0.07	0.03	0.13
Portal vein thrombosis	0.04	0.03	0.03	0.07	0.01	0.04	0.14	0.02	0.06	0.02	0.18
Sarcopenia	0.02	0.02	0.01	0.01	0.01	0.02	0.02	0.01	0.05	0.08	0.02
Malnutrition	0.07	0.07	0.06	0.08	0.03	0.07	0.06	0.05	0.15	0.04	0.08
Osteoporosis	0.02	0.05	0.04	0.01	0.10	0.05	0.06	0.02	0.05	0.00	0.02

Abbreviations: AIH = autoimmune hepatitis, ALD = alcohol‐related liver disease, BCS = Budd‐Chiari syndrome, Congestive = congestive hepatopathy, HBV = hepatitis B, HCV = hepatitis C, IQR = interquartile range, MASLD = metabolic dysfunction associated‐steatotic liver disease, PBC = primary biliary cholangitis, SD = standard deviation.

Patients with PBC were mostly female (81.81%), but this proportion was lower than expected. Autoimmune hepatitis (AIH) and WD were also mostly female (67.81% and 56.67%), whilst male patients made up the majority of all other aetiologies, with only 18.40% and 25.95% of HBV and ALD related admissions being female respectively. The proportion of AIH admissions which were female steadily decreased over the study period (80.55% in 2011 to 59.52% in 2022, Figure S3). No other trends in sex by aetiology were observed.

### 3.5 Duration of Hospital Admission, Complications of Cirrhosis and Inpatient Mortality by Aetiology

Admissions with ALD had a 9.04% risk of inpatient mortality over the study period. Ascites was the most common complication of liver disease in ALD, affecting 32.11%, followed by bacterial infections other than SBP (28.84%), hepatic encephalopathy (21.78%) and HCC (18.19%). Compared with the non‐ALD cohort, ALD had higher rates of all investigated complications apart from HCC, portal vein thrombosis (PVT) and—in recent years—HRS. The proportion and overall number of ALD with HRS reduced considerably from 2019 onwards (Figure S4). Trends in most other complication rates by year were similar across ALD and non‐ALD.

Both duration of hospital admission (9.49 days, SD = 15.90) and inpatient mortality (3.09%) were lowest in PBC (Table 2). Similarly, PBC displayed some of the lowest rates of all complications of liver disease apart from osteoporosis, where it displayed the highest as expected (10.3%) (Figure S5). Of note, haemochromatosis had relatively higher inpatient mortality rates (10.00%), durations of admission (15.82 days, SD = 26.05) and the second highest rates of HRS (11.11%), SBP (9.44%), portal vein thrombosis (13.89%) and osteoporosis (5.56%). It also had the third highest likelihood of HCC (26.67%).

Compared to other major aetiologies such as ALD, HBV and HCV, MASLD had lower proportions of almost every cirrhosis‐related complication. HBV had the highest proportion of HCC (46.42%), followed by HCV (27.72%).

### 3.6 Comparison of Cirrhosis With Other Major Chronic Health Care Admissions

#### 3.6.1 Demographic Data

**FIGURE 3:**
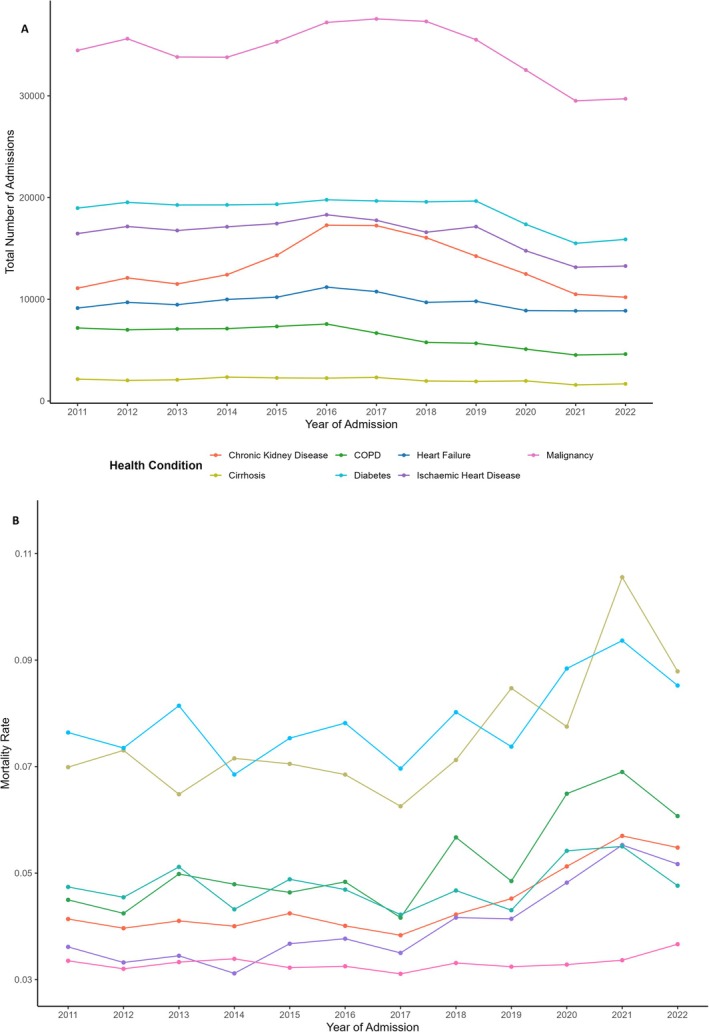
(A) Total number of admissions with various chronic health conditions per year. (B) Inpatient mortality rate for admissions with various chronic health conditions per year.

Table 3 demonstrates that patients with cirrhosis represented the lowest number of admissions over the study period (24,567) in comparison to the other analysed disease conditions. Like cirrhosis, there have been decreasing numbers of admissions with all other chronic health conditions investigated in recent years (Figure 3A). The proportion of patients with repeat presentations each year was highest in those with malignancy (47.26%) followed by cirrhosis (44.89%). The proportion of patients with repeat presentations has similarly decreased in recent years across all chronic health conditions (Figure S6).

**TABLE 3:** Demographics, length of admission and inpatient mortality rates of various chronic health conditions.

	Chronic health conditions						
	COPD	Diabetes	CCF	IHD	Malignancies	CKD	Cirrhosis
Total admissions	75,623	223,845	116,571	195,943	412,411	205,339	24,567
Age (median, IQR)	71	70	73	73	64	73	62
	63–70	60–77	63–80	64–79	53–74	62–80	53–70
Proportion female	0.41	0.41	0.38	0.30	0.47	0.41	0.35
Proportion of patients with repeat presentations	0.40	0.39	0.34	0.43	0.47	0.45	0.45
Days of admission (mean, (SD))	11.67	10.86	12.60	8.91	8.99	11.16	11.29
	16.88	16.40	17.67	13.34	13.33	15.71	17.46
Inpatient mortality rate	0.05	0.05	0.08	0.04	0.03	0.04	0.07
Age adjusted mortality rate (European standard)	0.03	0.03	0.07	0.03	0.02	0.03	0.06
Proportion of chronic health conditions with particular comorbidities							
COPD	—	0.10	0.18	0.14	0.06	0.13	0.07
Diabetes	0.31	—	0.35	0.35	0.16	0.35	0.36
CCF	0.27	0.18	—	0.31	0.05	0.27	0.10
IHD	0.36	0.31	0.54	—	0.09	0.36	0.11
Osteoporosis	0.05	0.02	0.03	0.02	0.01	0.03	0.03
CKD	0.26	0.25	0.37	0.29	0.12	—	0.17
Malignancy	0.24	0.23	0.13	0.15	—	0.23	0.30
Non‐hepatobiliary malignancy	0.23	0.22	0.13	0.14	—	0.23	0.13

Abbreviations: CCF = congestive cardiac failure, CKD = chronic kidney disease, COPD = chronic obstructive pulmonary disease, IHD = ischaemic heart disease, IQR = interquartile range.

**FIGURE 4:**
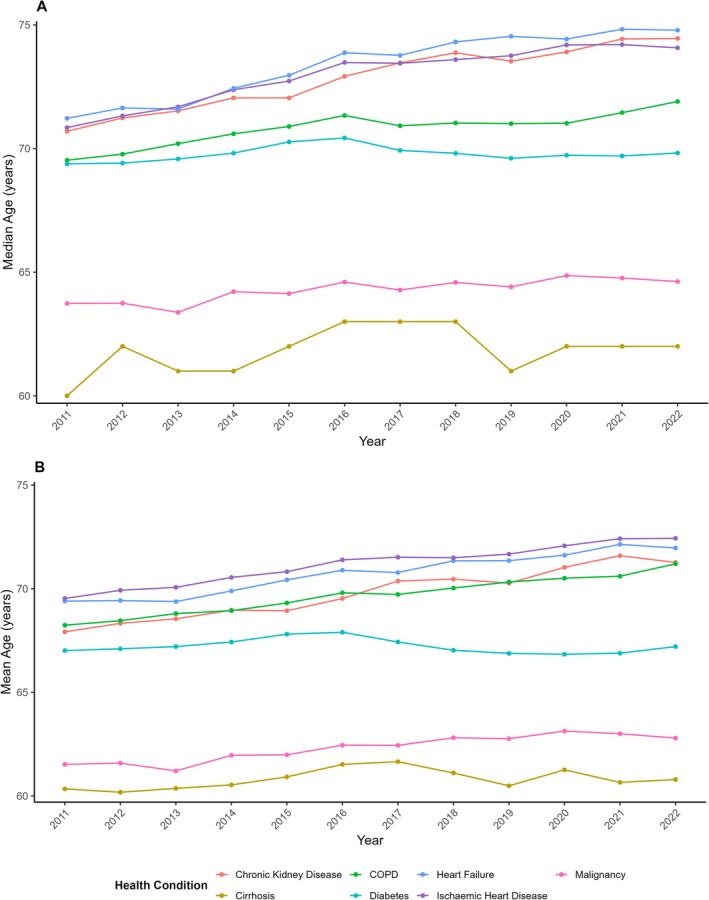
A) Median and B) Mean age at admission for various chronic health conditions per year.

The percentage of admissions which were female was lowest in IHD (30.02%), followed by cirrhosis (34.49%). The median age of cirrhosis patients was in general almost a decade younger than all other chronic health conditions, apart from malignancy where cirrhosis patients were still a median age of 2.32 years younger. All conditions other than diabetes have seen greater age increases over time compared to cirrhosis (Figure 4). Compared to other chronic health conditions, using quantile regression, cirrhosis experienced the second lowest age increase rate at the 25th percentile of age, the third lowest at the 50th percentile and the lowest rate by a considerable margin at the 75th (rate of age change by −0.03 per year compared to 0.27 to 0.43 for all other health conditions, Table S3).

Quantile regressions were further performed on subsets of the data, where cirrhosis was compared against other chronic health conditions, with the exclusion of patients who had both cirrhosis and the other chronic health condition being compared against (Figure S7). This demonstrated the age of patients with IHD, CCF, COPD and CKD has increased at a considerably higher rate than cirrhosis across each quartile. Patient age has seen the most profound increase at the 75th percentile in all health conditions apart from cirrhosis, which has increased the least.

#### 3.6.2 Length of Admission and Mortality

With regards to the duration of hospital admission, cirrhosis falls in the middle of the chronic health conditions, with a mean duration of 11.29 days (SD 17.46), compared to CCF with the highest (12.60 days, SD = 16.67) and IHD (8.91 days, SD = 13.34 days) and malignancy (8.99 days SD = 13.33) with the lowest. Over time, the duration of hospital admission has decreased from 2011 to the middle of the observation period, with increasing durations seen for cirrhosis, COPD and CKD since (Figure S7).

In‐hospital mortality by health condition was highest in CCF (7.91%) followed by cirrhosis (7.45%). When adjusted for age using the European standard, cirrhosis continued to have the second highest mortality at 6.49% compared to 6.96% for CCF. When directly comparing mortality rates on mutually exclusive subsets of data based on the condition being compared, cirrhosis had a considerably higher mortality rate than all other conditions being compared against apart from CCF (0.064 vs. 0.077, Table S4). In recent years, however, cirrhosis has overtaken CCF as the health condition associated with the highest inpatient mortality (Figure 3B).

Despite cirrhosis being associated with immune dysfunction, [18] admissions with cirrhosis experienced lower proportions of all types of bacterial infections, apart from peritonitis, than COPD, diabetes, CCF and CKD (Figure S9).

#### 3.6.3 Comorbidities

Patients with cirrhosis had the lowest rates of having COPD (7.43%), CCF (9.79%), IHD (10.63%) and CKD (17.42%) compared to all other chronic health conditions apart from malignancy (Table 3). Cirrhosis patients had the highest rates of malignancy (29.83%), but the lowest rates of non‐hepatobiliary malignancy (12.64%). Cirrhosis patients additionally had the highest incidence of diabetes (35.63%), but this was only marginally higher than CCF (35.30%), CKD (35.01%) and IHD (34.68%). 3.32% of cirrhosis patients had a diagnosis of osteoporosis, behind only COPD at 4.61%.

### 3.7 Hospital Admissions and Inpatient Mortality During the COVID‐19 Pandemic

As can be seen in Figures S10–S12: hospital admissions with cirrhosis reached their lowest level around December 2020 (112 per month), coinciding with a peak in inpatient deaths and inpatient mortality. A similar trend was seen for all other chronic health conditions studied, with monthly admissions reaching a low of 5724 in December, and the highest number of inpatient deaths throughout the observation period of 533. These trends coincided with the first major peak in COVID‐19 cases, hospitalisations and mortality in Germany.

In particular, the inpatient mortality rates per month reached their highest on record for every condition studied in December 2020, with CCF having the highest mortality rate of 15.81%, followed by cirrhosis (15.18%), COPD (14.15%) and diabetes (11.14%).

**FIGURE 5:**
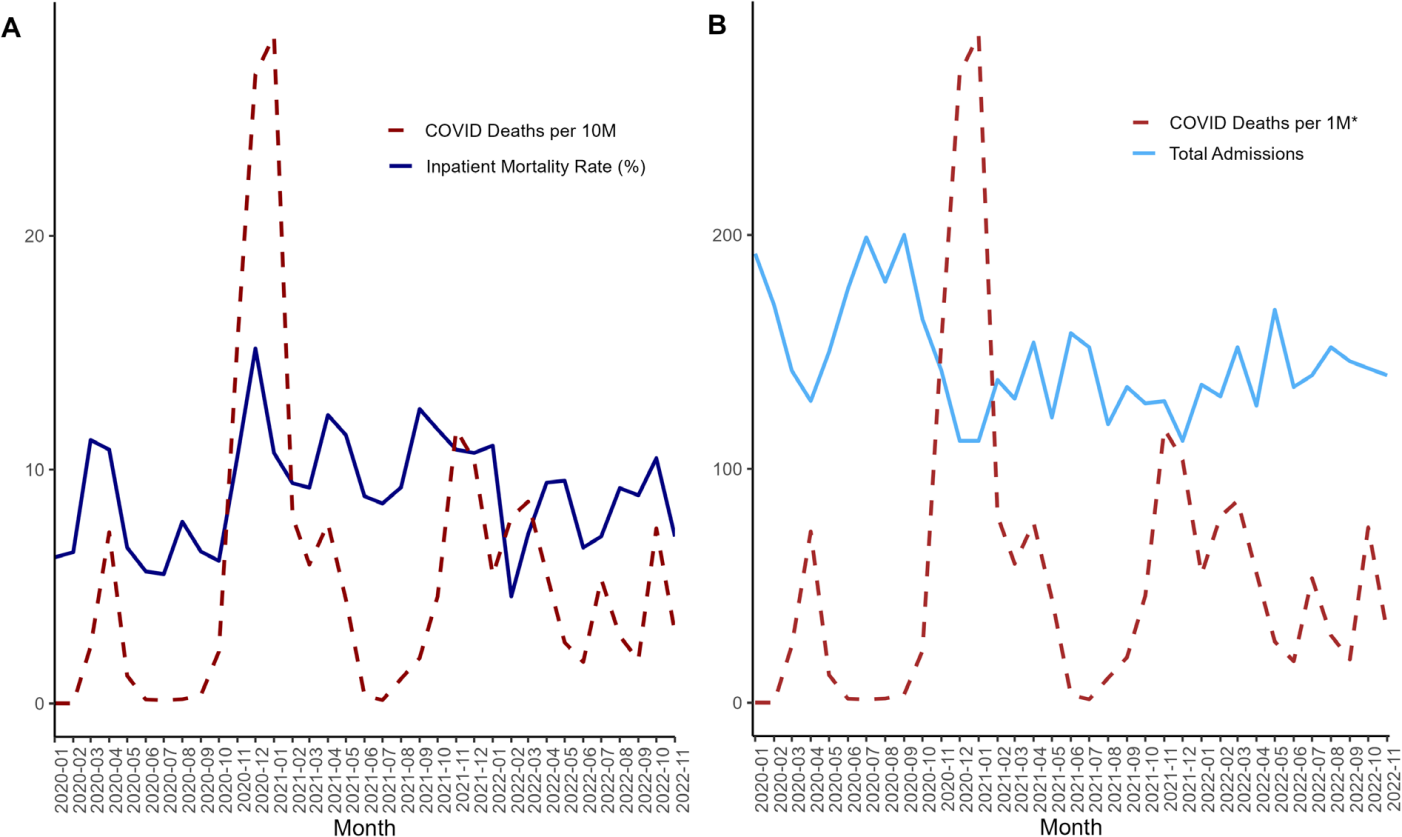
COVID deaths in Germany per month superimposed onto: (A) inpatient mortality rate of cirrhosis admissions per month and (B) cirrhosis admissions per month at The Charité.

Figure 5 suggests a positive relationship between monthly COVID‐19 deaths in Germany and the inpatient mortality rate for cirrhosis admissions at Charité. It also indicates an inverse relationship between monthly nationwide COVID‐19 deaths and the number of cirrhosis admissions. A similar trend was observed when considering COVID‐19 hospitalisations in Germany instead of deaths.

Ad‐hoc analysis using Spearman's correlation demonstrated moderate‐to‐strong relationships between monthly COVID‐19 deaths in Germany and monthly cirrhosis admissions (rho = −0.655, *p* < 0.001), as well as between monthly COVID‐19 deaths and monthly cirrhosis inpatient mortality (rho = 0.561, *p* < 0.001).

A median of 142 (IQR 131–154) cirrhosis admissions per month was noted during the COVID‐19 period, compared to 177 (IQR 166–191) admissions per month in the pre‐COVID era (*p* < 0.001). The monthly inpatient mortality rate during COVID‐19 was 9.24% (IQR 7.17–10.80), which was significantly higher than the rate of 7.03% (IQR 5.71–8.43) pre‐COVID‐19 (*p* < 0.001).

### 3.8 Validation Cohort—University Hospital, Leipzig

Key findings from our Charité University Hospital Berlin cohort were compared against a cohort of patients from University Hospital Leipzig, comprising 14,141 admissions from 2011 to 2022. An overview of admission numbers, characteristics, complications and mortality rates is summarised in Table S5. Both cohorts had a median age of 62, whilst the validation cohort comprised a lower proportion of female admissions (28% compared to 35%).

Over the years, ALD remained the most common underlying cause of cirrhosis, ranging from 49.67% to 63.98% of total admissions. There were fluctuating but largely stable rates of MASLD (ranging from 1.83% to 3.70%), with 2.52% of cirrhosis admissions having MASLD as an aetiology in 2022. HCV trended downwards from 5.64% in 2012 to 0.61% by 2022. Similarly, HBV reached a peak of 2.26% in 2015 and trended down towards 0.61% by 2022.

Ascites was the most common complication (36.52% of all admissions), followed by bacterial infections other than SBP (31.95%). Rates of ascites, HE, HRS, bacterial infections other than SBP, and malnutrition all peaked towards the middle of the observation period, then trended downwards thereafter, whereas rates of HCC and PVT peaked earlier. Rates of SBP, variceal haemorrhage and other GI bleeding remained stable over the years.

SBP and HRS were associated with the highest inpatient mortality rates of 40.06% and 36.21%, respectively. Over time, there were fluctuations in mortality rates associated with gastrointestinal bleeding; however, rising inpatient mortality was noted again in recent years for VH (38.13% in 2022) and other GI bleeding (34.87%).

Weak relationships were noted between monthly national COVID‐19 deaths and monthly admissions with cirrhosis (rho = −0.352, *p* = 0.035) and monthly inpatient mortality rates (rho = 0.320, *p* = 0.058). The median monthly cirrhosis admissions during the COVID‐19 era were 98 (IQR 90–106), which was the same as pre‐COVID‐19 (98, IQR 89–106). The median monthly inpatient mortality rate was 10.70% (IQR 8.00%–13.10%) during the COVID‐19 era, similar to the rate of 10.40% (IQR 8.08%–12.30%) pre‐COVID‐19.

## 4 Discussion

This study provides an up‐to‐date overview of the recent trends and burdens of cirrhosis in a large, metropolitan hospital network in Berlin, Germany. Over time, there has been a decrease in cirrhosis admissions in Berlin, but an increase in inpatient mortality rate, rate of complications and ICU admissions. Despite this, the overall numbers of inpatient deaths have remained relatively stable, and the incidence of most complications has been decreasing. This may suggest improved confidence in and access to outpatient and ambulatory care management of cirrhosis patients, meaning only the sickest patients are requiring hospitalisation.

These results are also likely to have been influenced by the recent establishment of a day‐care clinic at our institution, where patients are able to attend for procedures such as routine large volume paracenteses instead of requiring inpatient admission. The COVID pandemic has also likely impacted admission numbers and inpatient mortality rates. The spike in inpatient mortality in December 2020 for cirrhosis admissions is unlikely to be due to COVID‐19 deaths alone, as only 2 of the 17 deaths were COVID related for that month. The pandemic saw diversions in resources, ward closures, staffing shortages and a reluctance for patients to seek medical care, which may correspond with the fall in hospital admissions and rise in inpatient mortality from 2020 to 2021. These changes were less pronounced in the validation cohort. Compared to other similarly conducted studies in Western countries, our population had higher rates of HCC [19, 20, 21] and hepatic encephalopathy [19, 21]. In particular, compared to a cohort assessing admissions across all of Germany, our cohort had lower rates of PVT and ascites but considerably higher rates of HCC and bacterial infections [5]. This may be secondary to divergent demographics, aetiologies, or socioeconomic factors compared with the rest of the country, as well as our large HCC transplant program. The gross numbers of these complications, however, have been decreasing in recent years, perhaps reflecting the improved management of patients with cirrhosis over time.

There has been a considerable increase in the proportion and gross numbers of patients of sarcopenia, which may be secondary to the recent introduction of a dedicated ICD‐10‐GM code for sarcopenia, as well as improved recognition of this complication during the study period. The numbers of patients with SBP have not decreased in line with most other complications, the reason for which is not entirely clear. Patients with HRS and SBP persistently demonstrated the highest rates of inpatient mortality, as expected [22, 23], which was noted in the validation cohort as well. Interestingly, pneumonia was more common than SBP and was associated with a higher inpatient mortality, which may be due to the use of prophylactic antibiotics against SBP [24].

There has been a concerning rise in the mortality rate of admissions with variceal haemorrhage or gastrointestinal bleeding in general which needs to be reviewed. This may be secondary to the increased use of nonselective beta‐blockers for primary prophylaxis, [25] alongside the reduced presentations of variceal haemorrhage in recent years, leading to less interventionalist exposure to endoscopic management of gastrointestinal varices. Furthermore, reassuringly, total numbers of patients with VH, and to a lesser extent, other GI bleeding, appear to be decreasing in recent years. Alongside the higher MELD scores in these patients compared to previous years, the rising inpatient mortality associated with GI bleeding may instead be due to the underlying severity of our patients' liver disease.

The validation cohort (Leipzig) similarly demonstrated high inpatient mortality rates among patients with variceal and non‐variceal GI haemorrhage, and although there were higher rates in recent years, rates were fluctuating throughout the observation period.

There have also been shifts in the aetiologies of cirrhosis in recent years, although ALD has consistently been the most common cause, in line with most other Western countries [9]. There was a considerable decline in HCV in the early years of the study secondary to the introduction of direct acting antivirals [26], and this has persisted through the observation period. Even though HBV initially followed a similar trend, there have been increasing proportions and numbers of HBV admissions in recent years, perhaps secondary to changing demographics or immigration patterns which warrant further attention. In the validation cohort, this trend was not observed for HBV, possibly reflecting different demographic shifts between these cities in Germany.

There has been growing concern surrounding the increasing incidences of metabolic risk factors worldwide, and how this may lead to an increasing burden of MASLD as a cause for cirrhosis [9, 27]. In our cohort, however, there has not been a clear rise in cirrhosis secondary to MASLD, which is also in keeping with a decreasing proportion of metabolic risk factors such as diabetes and obesity in our patients over time. Reduced hepatic steatosis, which is a feature of advanced cirrhosis, would lead to greater difficulty in the diagnosis of MASLD, which may also be contributing to the unexpectedly low numbers. MASLD also displayed lower rates of complications of liver disease compared to other major aetiologies such as ALD and viral hepatitis, indicating the burden of MASLD is not yet as significant as was expected.

Of note, a higher proportion of PBC admissions were male than expected, with almost 19% being male compared to the expected 5%–10% in the general population [28, 29]. This suggests men with PBC may be more likely to be hospitalised or experience more severe disease than women [30]. There have also been rising proportions of males admitted with AIH over the study period—a trend seen elsewhere—perhaps suggesting an increased recognition of AIH in men over time [31].

The rates of complications of cirrhosis by aetiology in our cohort were largely in line with what was to be expected and known in the wider literature. Of note, PBC had the lowest rates of all complications apart from osteoporosis, [32] congestive hepatopathy had the highest rates of ascites, and the incidence of HCC was highest in HBV followed by HCV [33]. Of interest, HCH was associated with considerably higher rates of HCC and PVT, adding further evidence that iron overload may play a pathophysiological role in hepatic carcinogenesis and a prothrombotic state [34, 35].

Compared to other chronic health conditions investigated, there were concerning trends in the burdens of cirrhosis. Cirrhosis patients were among the highest rates of repeat presentations. They were considerably younger than patients with other conditions, and despite an overall aging population, cirrhosis patients were not ageing at a similar rate. This underpins the urgent need for improved management strategies and public health interventions to mitigate the impact of cirrhosis.

Despite being much younger and having fewer comorbidities, cirrhosis patients are still more likely to die in hospital compared to most other conditions, and this inpatient mortality rate has been increasing over time. In recent years, cirrhosis has overtaken heart failure as the condition with the highest inpatient mortality rate, despite being a median of 11 years younger and having lower rates of most comorbidities. The rates of osteoporosis were second only to COPD, which highlights the need to focus on optimising malnutrition and hypogonadism in the cirrhosis population [36].

This study highlights the changing burdens of cirrhosis and its complications and demonstrates how cirrhosis is falling behind other major health conditions regarding patient outcomes. As such, this study provides information which may guide improved resource allocation.

Limitations include the retrospective nature of this study and the possibilities of missing or incorrect data, in part due to the heavy reliance on ICD‐10‐GM for diagnoses. Codes and their recognition can change over time and rely heavily on discharge summary accuracy and coder competency. Despite this, studies have validated the use of ICD‐10 codes for identifying patients with cirrhosis and its complications [37, 38].

## 5 Conclusion

Cirrhosis remains a significant clinical and public health challenge. Although there have been some improvements over time, complication and mortality rates remain high, reflecting the need for enhanced inpatient care strategies. The higher burden of mortality without a relative increase in age in cirrhosis compared to other chronic conditions emphasises the need for targeted interventions and resource allocation. This study contributes to understanding the dynamic landscape of cirrhosis. Further research is needed to consolidate these findings.

## Author Contributions

**Julian Pohl:** conceptualization, methodology, software, formal analysis, data curation, writing – original draft, writing – review and editing, resources, visualization, investigation. **Nirbaanjot Walia:** conceptualization, methodology, software, data curation, investigation, formal analysis, visualization, resources, writing – original draft, writing – review and editing. **Lukas Hofmann:** software, data curation, resources. **Lena Wolters:** resources, data curation, software. **Martin Schulz:** data curation, resources, software. **Matthias Reinhardt:** data curation, methodology, software, supervision. **Brigitta Globke:** writing – review and editing, data curation. **Frank Tacke:** investigation, methodology, resources, writing – review and editing. **Thomas Berg:** data curation, resources, software. **Cornelius Engelmann:** writing – review and editing, project administration, supervision, funding acquisition, investigation.

## Disclosure

*Guarantor of the article*: Cornelius Engelmann.

## Conflicts of Interest

Julian Pohl has served as a speaker for Advanz. Thomas Berg has received research funding from Abbvie, Advanz, Gilead, Norgine, Orphalan and Sequana Medical. Thomas Berg has served as a speaker, a consultant and an advisory board member for Abbvie, Alexion, Albireo, Bayer, Gilead, GSK, Eisai, Intercept, Ipsen, Madrigal, Mirum, Orphalan and Sequana Medical. Thomas Berg has served as a speaker for Abbvie, Advance Pharma, Alexion, Bayer, Gilead, Eisai, Falk Foundation, Ipsen, and MedUpdate GmbH and Orphalan. Frank Tacke has served as a speaker, a consultant and an advisory board member for Gilead, AbbVie, Falk, Merz, Sanofi, Astra Zeneca, Orphalan, Boehringer, AstraZeneca, GSK, BMS, Ipsen, Pfizer, Novartis, Novo Nordisk, MSD, Sanofi and has received research funding from AstraZeneca, MSD, Gilead, Agomab. Cornelius Engelmann has served as a speaker, a consultant and an advisory board member for Boehringer Ingelheim, Alibreo/Ipsen, Gilead and has received research funding from European Union, Deutsche Forschungsgemeinschaft, Else Kröner Freseniusstiftung. Cornelius Engelmann owns stocks and shares in Hepxy Ltd. Cornelius Engelmann owns a patent for Stem‐cell mobilisation plus TLR4 inhibition to treat liver failure and for P21 expression in liver tissue as a prognostic marker in liver disease.

## Supporting information


Data S1:

## Data Availability

The data that support the findings of this study are available on request from the corresponding author. The data are not publicly available due to privacy or ethical restrictions.
