# Supplementary material for: Emerging Trends in Patients Hospitalised With Cirrhosis—Aetiologies, Complications and Outcomes Compared to Other Chronic Health Conditions
Source: Aliment Pharmacol Ther. 2025 Jul 28;62(11-12):1108–24. doi: 10.1111/apt.70287 (PMC12646628; doi:10.1111/apt.70287)
Supplement: Supplementary file 1 — Data S1: [file APT-62-1108-s001.docx]

**Supplementary Materials**

**Table S1: ICD-10-GM codes used**

| **Disease or complication** | ICD-10-GM |
| --- | --- |
| Cirrhosis | K70.3  K74.3 – K74.7 |
| **Aetiology** |  |
| Alcoholic Liver Disease | K70 |
| Metabolic Dysfunction Associated-Steatotic Liver Disease | K75.8 K76.0 |
| Hepatitis C | B18.2 |
| Hepatitis B | B18.0. B18.1 |
| Primary Biliary Cholangitis | K74.3 |
| Autoimmune Hepatitis | K75.3 |
| Hemochromatosis | E83.1 |
| Congestive Liver Disease | K76.1 |
| Toxic | K71 |
| Wilson’s Disease | E83.0 |
| Budd-Chiari Syndrome | I82.0 |
| **Complications** |  |
| Ascites | R18 |
| Hepatic Encephalopathy | K72.7 |
| Variceal Haemorrhage | I98.3 |
| Other GI bleeding | K92.0 – K92.2 |
| Hepatorenal Syndrome | K76.7 |
| Bacterial Peritonitis | K65 |
| Bacterial infection – other | A01 – A05 A2 – A4  B95. B96  G00. G01  J13 – J14  L00 – L03  M00  N30.0. N34. N39. N41.0. N45.0 |
| Hepatocellular Carcinoma | C22 |
| Portal Vein Thrombosis | I81 |
| Sarcopaenia | M62.50 |
| Osteoporosis | M80 |
| Malnutrition | E40-E46 |
| **Other Health Conditions** |  |
| Malignancy | C |
| Diabetes | E10 – E14 |
| Obesity | E65 – E68 |
| Ischaemic Heart Disease | I20 – I25 |
| Congestive Cardiac Failure | I50 |
| Chronic Obstructive Pulmonary Disease | J43 – J44 |
| Cerebrovascular Disease | I60 – I65 |
| Chronic Kidney Disease | N17 – N19 |
| COVID-19 | U07.1 |

**Table S2: Inpatient mortality rate by complication and year**

|  | **2011** | **2012** | **2013** | **2014** | **2015** | **2016** | **2017** | **2018** | **2019** | **2020** | **2021** | **2022** | **Total** |
| --- | --- | --- | --- | --- | --- | --- | --- | --- | --- | --- | --- | --- | --- |
| Ascites | 0.17 | 0.19 | 0.16 | 0.15 | 0.15 | 0.17 | 0.17 | 0.15 | 0.15 | 0.18 | 0.21 | 0.19 | 0.17 |
| Hepatic Encephalopathy | 0.19 | 0.26 | 0.22 | 0.25 | 0.21 | 0.21 | 0.24 | 0.22 | 0.23 | 0.21 | 0.32 | 0.23 | 0.23 |
| Variceal Haemorrhage | 0.16 | 0.20 | 0.17 | 0.19 | 0.15 | 0.27 | 0.26 | 0.26 | 0.30 | 0.31 | 0.30 | 0.28 | 0.23 |
| Other Gastrointestinal Bleeding | 0.11 | 0.20 | 0.30 | 0.22 | 0.21 | 0.12 | 0.26 | 0.30 | 0.37 | 0.28 | 0.39 | 0.31 | 0.24 |
| Hepatorenal Syndrome | 0.28 | 0.35 | 0.32 | 0.32 | 0.32 | 0.34 | 0.30 | 0.34 | 0.34 | 0.37 | 0.41 | 0.35 | 0.33 |
| Bacterial Peritonitis | 0.37 | 0.37 | 0.34 | 0.29 | 0.29 | 0.35 | 0.32 | 0.35 | 0.33 | 0.31 | 0.37 | 0.31 | 0.33 |
| Other Bacterial Infections | 0.23 | 0.24 | 0.22 | 0.22 | 0.20 | 0.19 | 0.18 | 0.21 | 0.26 | 0.22 | 0.26 | 0.24 | 0.22 |
| Hepatocellular Carcinoma | 0.04 | 0.05 | 0.04 | 0.07 | 0.07 | 0.06 | 0.03 | 0.04 | 0.06 | 0.05 | 0.08 | 0.07 | 0.05 |
| Portal Vein Thrombosis | 0.13 | 0.10 | 0.14 | 0.12 | 0.10 | 0.17 | 0.09 | 0.11 | 0.24 | 0.21 | 0.21 | 0.12 | 0.14 |
| Sarcopenia | NA | NA | NA | NA | NA | 0.24 | 0.16 | 0.17 | 0.27 | 0.17 | 0.35 | 0.19 | 0.21 |
| Malnutrition | 0.09 | 0.08 | \| 0.07 \| \| --- \| | 0.12 | 0.12 | 0.13 | 0.16 | 0.15 | 0.15 | 0.12 | 0.20 | 0.16 | 0.13 |
| Osteoporosis | 0.05 | 0.05 | 0.04 | 0.08 | 0.03 | 0.07 | 0.09 | 0.06 | 0.00 | 0.00 | 0.11 | 0.22 | 0.06 |

**Table S3: Quantile Regression - Change in Age per Year in Various Chronic Health Conditions at the 25th. 50th and 75th Percentiles**

| **Condition. percentile** |  |  | **95% Confidence Interval** | | **n** |
| --- | --- | --- | --- | --- | --- |
| **Congestive Cardiac Failure** | **Coefficient** | ***p*** | Lower | Upper | 113585 |
| 0.25 | 0.22 | 0.00 | 0.18 | 0.27 |  |
| 0.5 | 0.38 | 0.00 | 0.32 | 0.43 |  |
| 0.75 | 0.41 | 0.00 | 0.38 | 0.44 |  |
| **Ischaemic Heart Disease** |  |  |  |  | 195804 |
| 0.25 | 0.18 | 0.00 | 0.14 | 0.22 |  |
| 0.5 | 0.32 | 0.00 | 0.29 | 0.36 |  |
| 0.75 | 0.41 | 0.00 | 0.38 | 0.44 |  |
| **Chronic Obstructive Pulmonary Disease** | |  |  |  | 75270 |
| 0.25 | 0.23 | 0.00 | 0.20 | 0.27 |  |
| 0.5 | 0.19 | 0.00 | 0.15 | 0.22 |  |
| 0.75 | 0.34 | 0.00 | 0.31 | 0.36 |  |
| **Malignancy** |  |  |  |  | 393870 |
| 0.25 | 0.21 | 0.00 | 0.18 | 0.24 |  |
| 0.5 | 0.11 | 0.00 | 0.08 | 0.15 |  |
| 0.75 | 0.27 | 0.00 | 0.24 | 0.29 |  |
| **Chronic Kidney Disease** |  |  |  |  | 157476 |
| 0.25 | 0.28 | 0.00 | 0.22 | 0.34 |  |
| 0.5 | 0.36 | 0.00 | 0.32 | 0.41 |  |
| 0.75 | 0.43 | 0.00 | 0.40 | 0.46 |  |
| **Diabetes** |  |  |  |  | 220909 |
| 0.25 | -0.11 | 0.00 | -0.14 | -0.08 |  |
| 0.5 | 0.03 | 0.01 | 0.00 | 0.05 |  |
| 0.75 | 0.31 | 0.00 | 0.28 | 0.33 |  |
| **Cirrhosis** |  |  |  |  | 24567 |
| 0.25 | 0.10 | 0.01 | 0.03 | 0.18 |  |
| 0.5 | 0.15 | 0.00 | 0.10 | 0.20 |  |
| 0.75 | -0.03 | 0.31 | -0.08 | 0.02 |  |

**Table S4: Inpatient mortality rate for admissions with cirrhosis compared to other chronic health conditions (mutually exclusive comparisons)**

| **Cirrhosis** | **Mortality Rate (%)** | **Condition** | **Mortality Rate (%)** |
| --- | --- | --- | --- |
|  | 7.17% | COPD | 4.93% |
|  | 6.64% | Diabetes | 4.61% |
|  | 6.37% | CCF | 7.71% |
|  | 7.14% | IHD | 3.88% |
|  | 7.73% | Malignancy | 3.34% |
|  | 6.99% | CKD | 4.28% |

Performed on subsets of data. where admissions with both cirrhosis and the chronic health condition being compared against are excluded.
**Abbreviations**: COPD = Chronic Obstructive Pulmonary Disease; CCF = Congestive Cardiac Failure; IHD = Ischaemic Heart Disease; CKD = Chronic Kidney Disease; IQR = Interquartile Range; SD = Standard Deviation.

**Table S5: Validation Cohort – University Hospital Leipzig - Characteristics and complications of patients admitted with cirrhosis**

**Abbreviations**: MASLD = Metabolic Dysfunction-Associated Steatotic Liver Disease; BCS = Budd-Chiari Syndrome; IQR = Interquartile Range; SD = Standard Deviation

|  | **2011** | **2012** | **2013** | **2014** | **2015** | **2016** | **2017** | **2018** | **2019** | **2020** | **2021** | **2022** | **Total** |
| --- | --- | --- | --- | --- | --- | --- | --- | --- | --- | --- | --- | --- | --- |
| **Admissions** | 1092 | 1224 | 1130 | 1163 | 1152 | 1243 | 1218 | 1256 | 1152 | 1216 | 1145 | 1150 | 14141 |
| **In-hospital mortality (n)** | 113 | 116 | 139 | 125 | 119 | 129 | 125 | 144 | 104 | 112 | 126 | 135 | 1487 |
| **In-hospital mortality rate** | 0.10 | 0.09 | 0.12 | 0.11 | 0.10 | 0.10 | 0.10 | 0.11 | 0.09 | 0.09 | 0.11 | 0.12 | 0.11 |
| **Age (median years. IQR))** | 58  (51–66) | 60  (52–68) | 61  (54–67) | 62  (54–68) | 62  (54–67) | 62  (55–68) | 62  (53–68) | 62  (55–68) | 63  (57–69) | 62  (54–69) | 63  (55–69) | 63  (56–70) | 62  (54–68) |
| **Days of admission (mean (SD))** | 13.5 (19.4) | 12.3 (18.2) | 11.6 (15.4) | 10.8 (15.2) | 10.7 (15.1) | 11.2 (15.4) | 11.0 (14.8) | 12.0 (18.1) | 11.4 (15.7) | 10.6 (14.7) | 11.2 (17.4) | 11.7 (17.3) | 11.5 (16.5) |
| **Sex (% female)** | 26.6 | 28.5 | 27.8 | 25.8 | 23.4 | 27 | 31.4 | 34 | 29.2 | 28.6 | 30.3 | 26.2 | 28.3% |
| **Aetiology (n (% of total admissions)** | | | | | | | | | | | | | |
| Alcohol-related Liver Disease | 686 (62.8%) | 782 (63.9%) | 723  (64%) | 689 (59.2%) | 654 (56.8%) | 644 (51.8%) | 605 (49.7%) | 670 (53.3%) | 606 (52.6%) | 690 (56.7%) | 627 (54.8%) | 698 (60.7%) | 8074 (57.1%) |
| MASLD | 26 (2.4%) | 32 (2.6%) | 23 (2%) | 29 (2.5%) | 33 (2.9%) | 46 (3.7%) | 39 (3.2%) | 23 (1.8%) | 30 (2.6%) | 29 (2.4%) | 36 (3.1%) | 29 (2.5%) | 375 (2.7%) |
| Hepatitis C | 31 (2.8%) | 69 (5.6%) | 63 (5.6%) | 58 (5%) | 32 (2.8%) | 46 (3.7%) | 40 (3.3%) | 17 (1.4%) | 19 (1.6%) | 11 (0.9%) | 11 (1%) | 7 (0.6%) | 404 (2.9%) |
| Hepatitis B | 19 (1.7%) | 23 (1.9%) | 8 (0.7%) | 19 (1.6%) | 26 (2.3%) | 26 (2.1%) | 18 (1.5%) | 7 (0.6%) | 17 (1.5%) | 10 (0.8%) | 7 (0.6%) | 7 (0.6%) | 187 (1.3%) |
| Primary Biliary Cholangitis | 45 (4.1%) | 47 (3.8%) | 29 (2.6%) | 30 (2.6%) | 33 (2.9%) | 40 (3.2%) | 37 (3%) | 53 (4.2%) | 67 (5.8%) | 31 (2.5%) | 24 (2.1%) | 22 (1.9%) | 458 (3.2%) |
| Autoimmune Hepatitis | 13 (1.2%) | 23 (1.9%) | 12 (1.1%) | 7 (0.6%) | 12 (1%) | 17 (1.4%) | 23 (1.9%) | 11 (0.9%) | 1 (0.1%) | 10 (0.8%) | 17 (1.5%) | 14 (1.2%) | 160 (1.3%) |
| Haemachromatosis | 4 (0.4%) | 10 (0.8%) | 3 (0.3%) | 10 (0.9%) | 13 (1.1%) | 6 (0.5%) | 10 (0.8%) | 7 (0.6%) | 3 (0.3%) | 4 (0.3%) | 4 (0.3%) | 1 (0.1%) | 75 (0.5%) |
| Congestive Hepatopathy | 3 (0.3%) | 4 (0.3%) | 8 (0.7%) | 5 (0.4%) | 6 (0.5%) | 9 (0.7%) | 7 (0.6%) | 8 (0.6%) | 6 (0.5%) | 11 (0.9%) | 3 (0.3%) | 3 (0.3%) | 73 (0.5%) |
| Toxic | 10 (0.9%) | 10 (0.8%) | 8 (0.7%) | 7 (0.6%) | 4 (0.3%) | 11 (0.9%) | 10 (0.8%) | 3 (0.2%) | 4 (0.3%) | 3 (0.2%) | 9 (0.8%) | 2 (0.2%) | 81 (0.6%) |
| Wilsons | 6 (0.5%) | 1 (0.1%) | 4 (0.4%) | 7 (0.6%) | 7 (0.6%) | 2 (0.2%) | 7 (0.6%) | 5 (0.4%) | 3 (0.3%) | 2 (0.2%) | 5 (0.4%) | 4 (0.3%) | 53 (0.4%) |
| BCS | 6 (0.5%) | 6 (0.5%) | 6 (0.5%) | 3 (0.3%) | 18 (1.6%) | 5 (0.4%) | 5 (0.4%) | 2 (0.2%) | 7 (0.6%) | 3 (0.2%) | 0 | 0 | 61 (0.4%) |
| Undefined/Other | 276 (25.3%) | 267 (21.8%) | 281 (24.9%) | 340 (29.2%) | 351 (30.5%) | 441 (35.5%) | 456 (37.4%) | 475 (37.8%) | 413 (35.9%) | 439 (36.1%) | 434 (37.9%) | 379  (33%) | 4680 (33.1%) |
| **Complications (n (% of total admissions)** | | | | | | | | | | | | | |
| Ascites | 328  (30%) | 436 (35.6%) | 393 (34.8%) | 371 (31.9%) | 425 (36.9%) | 465 (37.4%) | 466 (38.3%) | 516 (41.1%) | 448 (38.9%) | 487  (40%) | 423 (36.9%) | 409 (35.6%) | 5167 (36.5%) |
| Hepatic Encephalopathy | 39 (3.6%) | 145 (11.8%) | 118 (10.4%) | 118 (10.1%) | 171 (14.8%) | 202 (16.3%) | 258 (21.2%) | 310 (24.7%) | 238 (20.7%) | 215 (17.7%) | 165 (14.4%) | 146 (12.7%) | 2125 (15%) |
| Variceal Haemorrhage | 35 (3.2%) | 57 (4.7%) | 44 (3.9%) | 35 (3%) | 36 (3.1%) | 27 (2.2%) | 38 (3.1%) | 46 (3.7%) | 30 (2.6%) | 29 (2.4%) | 39 (3.4%) | 42 (3.7%) | 458 (3.2%) |
| Other Gastrointestinal Bleeding | 41 (3.8%) | 32 (2.6%) | 34 (3%) | 32 (2.8%) | 53 (4.6%) | 30 (2.4%) | 38 (3.1%) | 47 (3.7%) | 40 (3.5%) | 42 (3.5%) | 37 (3.2%) | 43 (3.7%) | 469 (3.3%) |
| Hepatorenal Syndrome | 69 (6.3%) | 94 (7.7%) | 91 (8.1%) | 79 (6.8%) | 109 (9.5%) | 99 (8%) | 127 (10.4%) | 119 (9.5%) | 89 (7.7%) | 111 (9.1%) | 80 (7%) | 95 (8.3%) | 1162 (8.2%) |
| Bacterial Peritonitis | 68 (6.2%) | 100 (8.2%) | 94 (8.3%) | 85 (7.3%) | 83 (7.2%) | 88 (7.1%) | 81 (6.7%) | 97 (7.7%) | 56 (4.9%) | 71 (5.8%) | 65 (5.7%) | 97 (8.4%) | 985 (7%) |
| Other Bacterial Infections | 229 (21%) | 244 (19.9%) | 290 (25.7%) | 341 (29.3%) | 383 (33.2%) | 465 (37.4%) | 476 (39.1%) | 489 (38.9%) | 435 (37.8%) | 422 (34.7%) | 371 (32.4%) | 373 (32.4%) | 4518 (31.9%) |
| Hepatocellular Carcinoma | 198 (18.1%) | 332 (27.1%) | 328 (29%) | 355 (30.5%) | 295 (25.6%) | 291 (23.4%) | 266 (21.8%) | 278 (22.1%) | 245 (21.3%) | 213 (17.5%) | 238 (20.8%) | 195 (17%) | 3234 (22.9%) |
| Portal Vein Thrombosis | 51 (4.7%) | 73 (6%) | 67 (5.9%) | 69 (5.9%) | 75 (6.5%) | 44 (3.5%) | 62 (5.1%) | 61 (4.9%) | 58 (5%) | 36 (3%) | 40 (3.5%) | 36 (3.1%) | 672 (4.8%) |
| Malnutrition | 4 (0.4%) | 5 (0.4%) | 21 (1.9%) | 15 (1.3%) | 86 (7.5%) | 244 (19.6%) | 221 (18.1%) | 176 (14%) | 98 (8.5%) | 72 (5.9%) | 42 (3.7%) | 45 (3.9%) | 1029 (7.3%) |
| **Inpatient mortality rate by complication** | | | | | | | | | | | | | |
| Ascites | 14.3% | 14.4% | 15.5% | 11.9% | 13.6% | 13.5% | 15% | 18% | 11.6% | 13.8% | 18.2% | 23.7% | 15.3% |
| Hepatic Encephalopathy | 25.6% | 21.4% | 28.8% | 18.6% | 25.7% | 27.2% | 22.1% | 27.1% | 24.4% | 18.1% | 32.7% | 41.8% | 25.8% |
| Variceal Haemorrhage | 22.9% | 35.1% | 47.7% | 34.3% | 33.3% | 48.1% | 34.2% | 28.3% | 23.3% | 44.8% | 46.2% | 38.1% | 36.2% |
| Other Gastrointestinal Bleeding | 19.5% | 18.8% | 29.4% | 40.6% | 22.6% | 16.7% | 31.6% | 36.2% | 25% | 35.7% | 32.4% | 34.9% | 28.8% |
| Hepatorenal Syndrome | 42% | 37.2% | 40.7% | 29.1% | 36.7% | 41.4% | 33.9% | 34.5% | 27% | 27.9% | 42.5% | 45.3% | 36.2% |
| Bacterial Peritonitis | 35.3% | 36% | 40.4% | 38.8% | 34.9% | 31.8% | 37% | 50.5% | 26.8% | 42.3% | 49.2% | 52.6% | 40.1% |
| Other Bacterial Infections | 26.6% | 27.9% | 33.4% | 26.1% | 23.8% | 22.4% | 22.3% | 23.5% | 20.2% | 22% | 28% | 29.5% | 24.9% |
| Hepatocellular Carcinoma | 7.1% | 6.9% | 7.9% | 7.3% | 5.4% | 3.4% | 4.5% | 7.6% | 3.7% | 5.2% | 5.5% | 11.3% | 6.3% |
| Portal Vein Thrombosis | 13.7% | 9.6% | 14.9% | 5.8% | 10.7% | 15.9% | 19.4% | 18% | 15.5% | 22.2% | 15% | 27.8% | 14.7% |
| Malnutrition | NA | 20% | 19% | 40% | 22.1% | 18.9% | 21.7% | 17.6% | 11.2% | 8.3% | 23.8% | 22.2% | 18.7% |
| **Comorbidities (n (% proportion of total admissions))** | | | | | | | | | | | | | |
| Diabetes | 355 (32.5%) | 431 (35.2%) | 347 (30.7%) | 411 (35.3%) | 430 (37.3%) | 477 (38.4%) | 480 (39.4%) | 524 (41.7%) | 503 (43.7%) | 503 (41.4%) | 417 (36.4%) | 414 (36%) | 5292 (37.4%) |
| Obesity | 82 (7.5%) | 80 (6.5%) | 59 (5.2%) | 73 (6.3%) | 91 (7.9%) | 154 (12.4%) | 152 (12.5%) | 132 (10.5%) | 155 (13.5%) | 165 (13.6%) | 129 (11.3%) | 89 (7.7%) | 1361 (9.6%) |
| Ischaemic Heart Disease | 68 (6.2%) | 62 (5.1%) | 56 (5%) | 88 (7.6%) | 97 (8.4%) | 102 (8.2%) | 119 (9.8%) | 150 (11.9%) | 140 (12.2%) | 115 (9.5%) | 113 (9.9%) | 98 (8.5%) | 1208 (8.5%) |
| Congestive Cardiac Failure | 67 (6.1%) | 73 (6%) | 46 (4.1%) | 95 (8.2%) | 114 (9.9%) | 122 (9.8%) | 180 (14.8%) | 191 (15.2%) | 167 (14.5%) | 142 (11.7%) | 88 (7.7%) | 102 (8.9%) | 1387 (9.8%) |
| Chronic Obstructive Pulmonary Disease | 67 (6.1%) | 82 (6.7%) | 67 (5.9%) | 55 (4.7%) | 65 (5.6%) | 80 (6.4%) | 74 (6.1%) | 84 (6.7%) | 69 (6%) | 68 (5.6%) | 64 (5.6%) | 55 (4.8%) | 830 (5.9%) |
| Chronic Kidney Disease | 232 (21.2%) | 256 (20.9%) | 284 (25.1%) | 283 (24.3%) | 386 (33.5%) | 474 (38.1%) | 537 (44.1%) | 570 (45.4%) | 541 (47%) | 436 (35.9%) | 400 (34.9%) | 362 (31.5%) | 4761 (33.7%) |
| Malignancy | 327 (29.9%) | 422 (34.5%) | 404 (35.8%) | 461 (39.6%) | 369 (32%) | 400 (32.2%) | 367 (30.1%) | 403 (32.1%) | 367 (31.9%) | 324 (26.6%) | 342 (29.9%) | 298 (25.9%) | 4484 (31.7%) |
| Cerebrovascular disease | 22 (2%) | 28 (2.3%) | 29 (2.6%) | 26 (2.2%) | 29 (2.5%) | 49 (3.9%) | 49 (4%) | 39 (3.1%) | 37 (3.2%) | 37 (3%) | 41 (3.6%) | 30 (2.6%) | 416 (2.9%) |

**Table S6: Association between Gastrointestinal Bleeding associated Mortality and MELD**

**Abbreviations**: MELD = Model of End-Stage Liver Disease

|  | **2011** | **2012** | **2013** | **2014** | **2015** | **2016** | **2017** | **2018** | **2019** | **2020** | **2021** | **2022** |
| --- | --- | --- | --- | --- | --- | --- | --- | --- | --- | --- | --- | --- |
| **Variceal Haemorrhage** | | | | | | | | | | | | |
| **Median MELD** | 11.9 | 14.3 | 15.0 | 16.1 | 15.5 | 16.4 | 16.2 | 14.2 | 15.6 | 17.6 | 17.8 | 17.3 |
| **Mortality (%)** | 15.5 | 20.0 | 16.8 | 18.6 | 15.2 | 27.0 | 26.4 | 25.7 | 30.4 | 31.4 | 30.4 | 27.7 |
| **Other Gastrointestinal Bleedings** | | | | | | | | | | | | |
| **Median MELD** | 11.6 | 11.8 | 17.2 | 15.4 | 15.0 | 15.6 | 17.5 | 15.1 | 16.9 | 15.8 | 16.3 | 19.3 |
| **Mortality (%)** | 10.5 | 20.5 | 30.0 | 21.5 | 21.1 | 11.8 | 25.7 | 29.5 | 36.7 | 27.9 | 39.1 | 31.3 |
| **Variceal Haemorrhage and Other Gastrointestinal Bleedings** | | | | | | | | | | | | |
| **Median MELD** | 11.9 | 13.6 | 15.1 | 15.7 | 15.3 | 15.7 | 17.0 | 14.8 | 16.4 | 17.0 | 17.5 | 18.6 |
| **Mortality (%)** | 12.6 | 20.2 | 21.1 | 19.2 | 18.6 | 18.8 | 25.7 | 27.2 | 32.6 | 30.0 | 34.5 | 29.5 |

**Table S7: TIPS in patients with variceal haemorrhage**

**Abbreviations**: VH = variceal haemorrhage

|  | **2011** | **2012** | **2013** | **2014** | **2015** | **2016** | **2017** | **2018** | **2019** | **2020** | **2021** | **2022** |
| --- | --- | --- | --- | --- | --- | --- | --- | --- | --- | --- | --- | --- |
| **Variceal Haemorrhage** | | | | | | | | | | | | |
| **Total (n)** | 103 | 85 | 95 | 118 | 79 | 89 | 72 | 70 | 92 | 86 | 79 | 65 |
| **TIPS (n)** | 12 | 10 | 8 | 10 | 6 | 8 | 6 | 5 | 5 | 4 | 11 | 6 |
| **TIPS in VH (%)** | 11.7 | 11.8 | 8.4 | 8.5 | 7.6 | 9.0 | 8.3 | 7.1 | 5.4 | 4.7 | 13.9 | 9.2 |
| **Mortality VH (%)** | 15.5 | 20.0 | 16.8 | 18.6 | 15.2 | 27.0 | 26.4 | 25.7 | 30.4 | 31.4 | 30.4 | 27.7 |


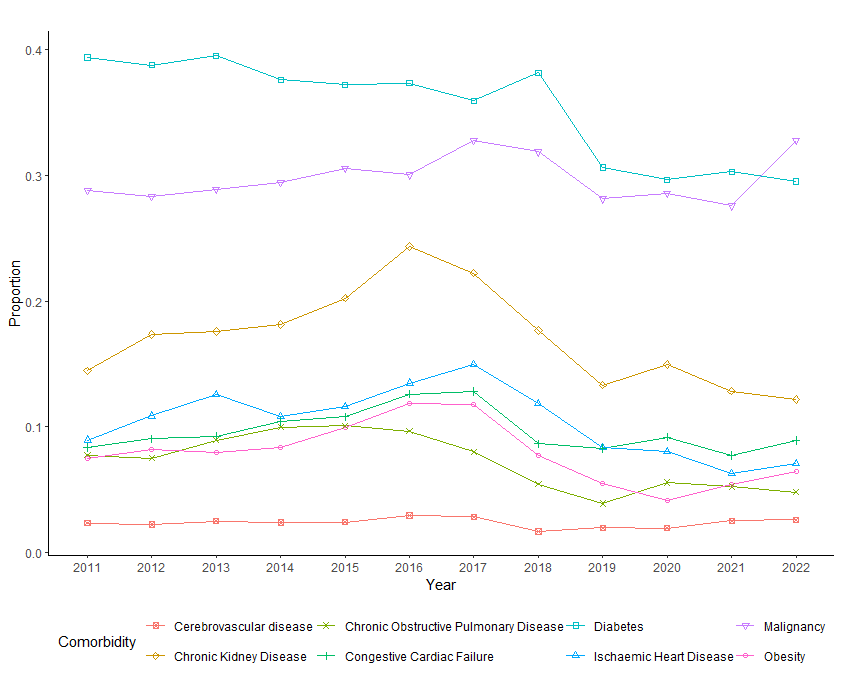


**Fig. S1: Proportion of cirrhosis admissions with other comorbidities**

**
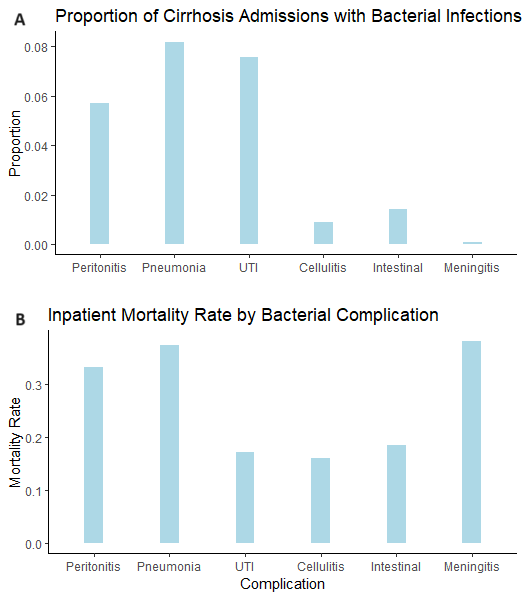
**

**Fig. S2: A) Proportion of cirrhosis admissions with bacterial infections. B) Inpatient mortality rate by bacterial complications**


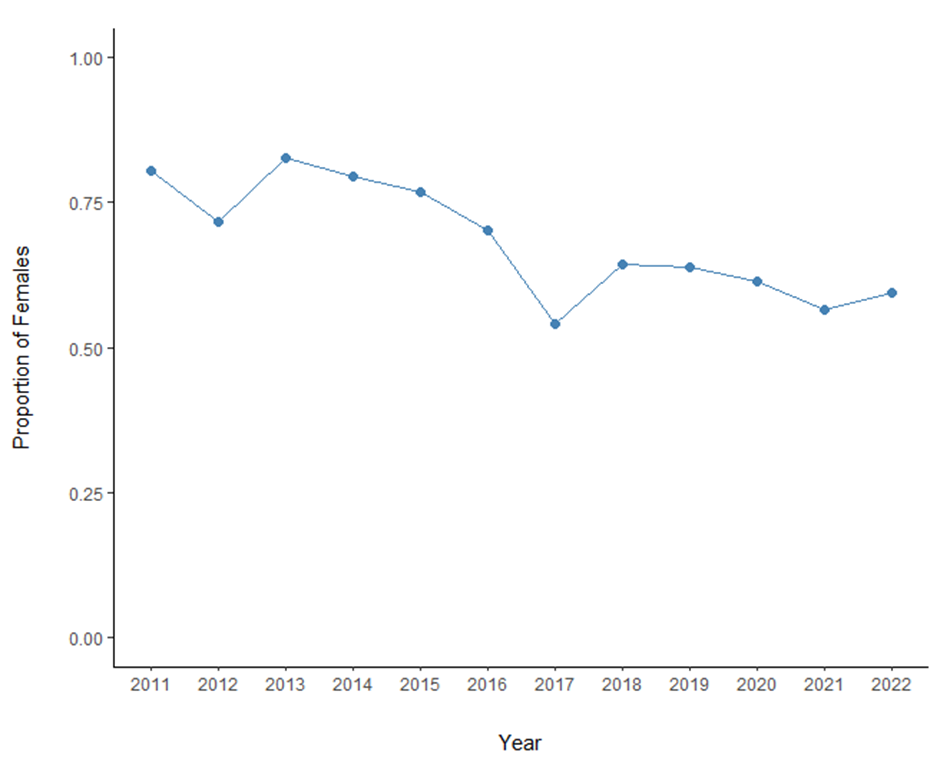


**Fig. S3: Proportion of autoimmune hepatitis admissions which were female per year**

**
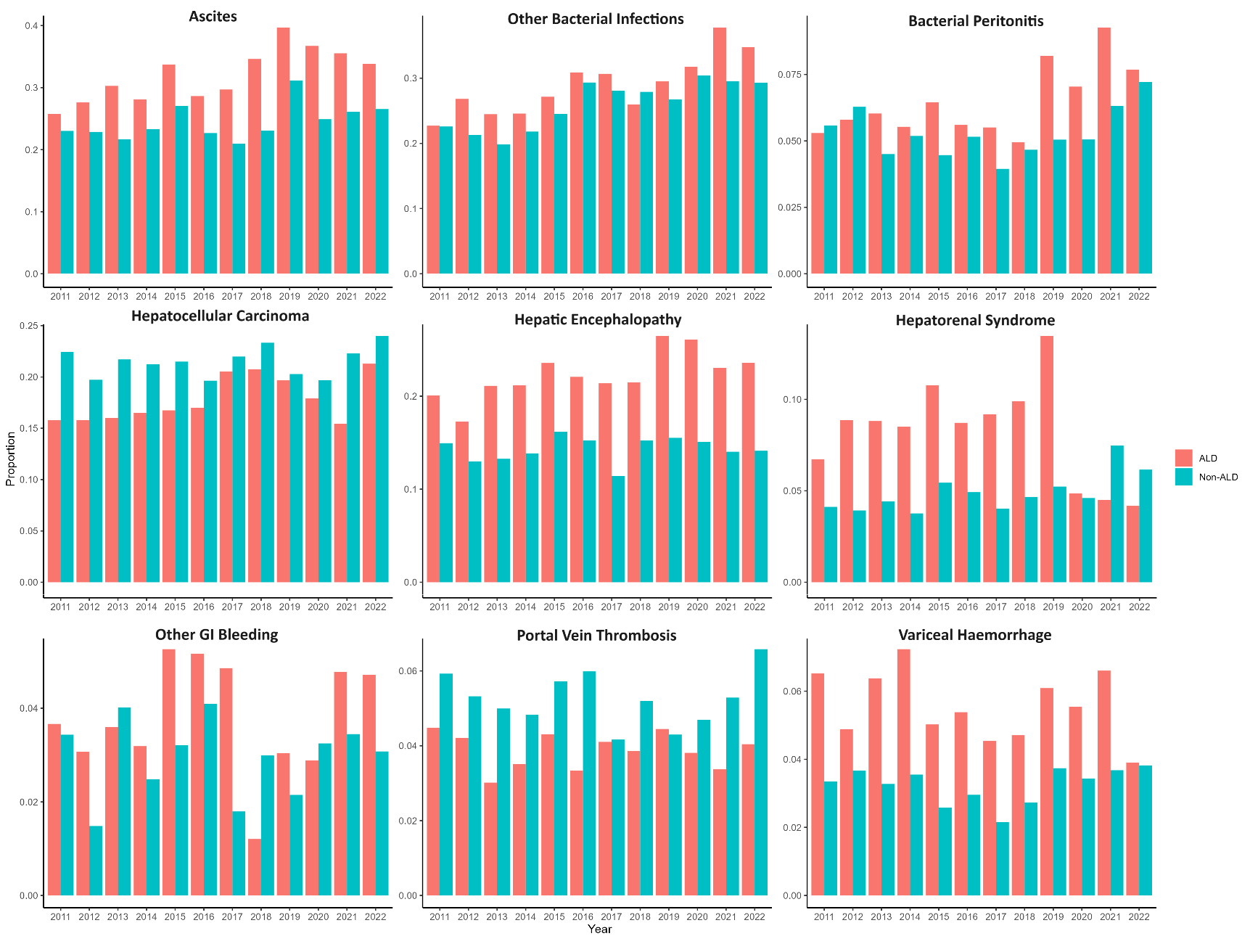
Fig. S4: Proportion of cirrhosis admissions with various complications by year in Alcohol-related Liver Disease and non-Alcohol-related Liver Disease**

**
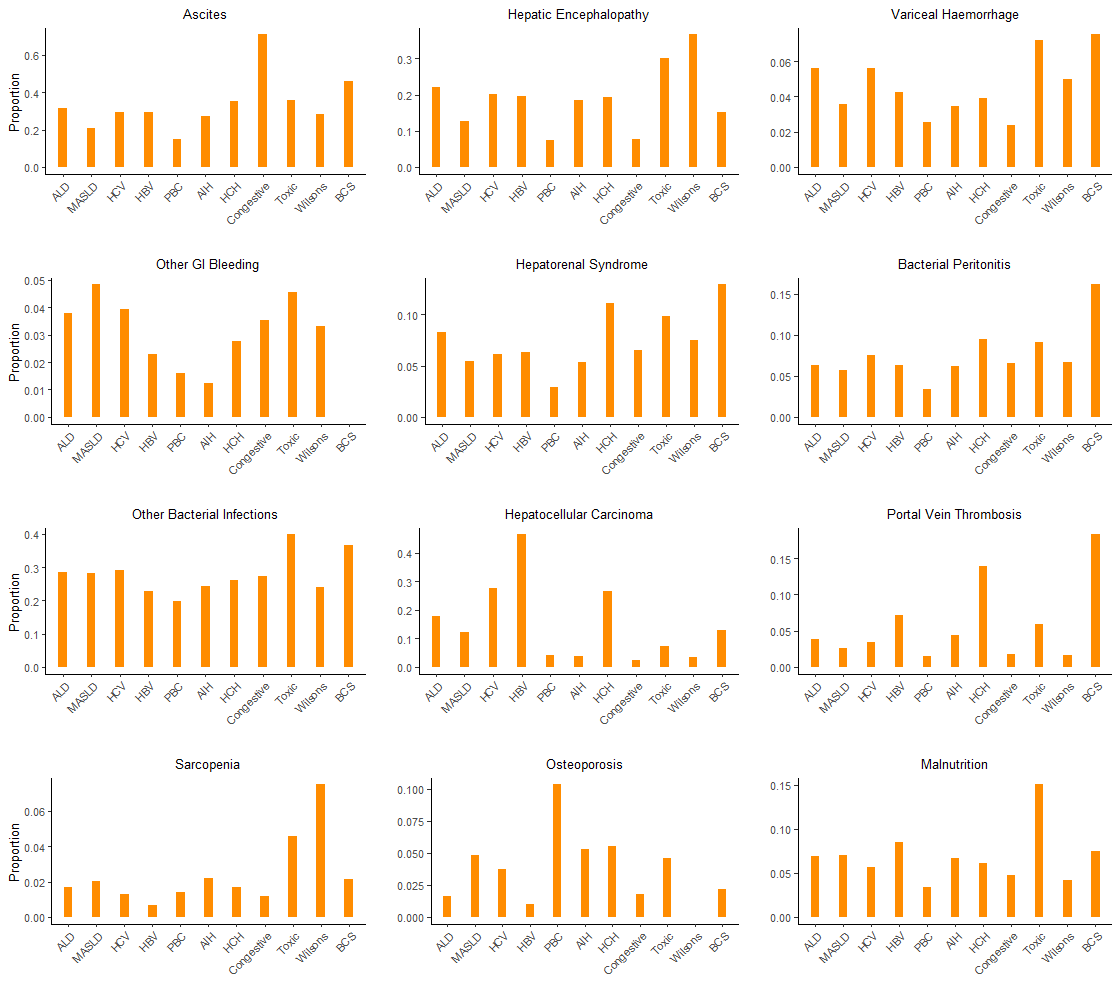
**

**Fig. S5 Proportion of admissions with a complication of liver disease by aetiology
Abbreviations:** ALD = Alcohol-related Liver Disease; MASLD = Metabolic Dysfunction Associated-Steatotic Liver Disease; HCV = Hepatitis C; HBV = Hepatitis B; PBC = Primary Biliary Cholangitis AIH = Autoimmune Hepatitis; HCH = Haemachromatosis; Congestive = Congestive Liver Disease; BCS = Budd-Chiari Syndrome

**
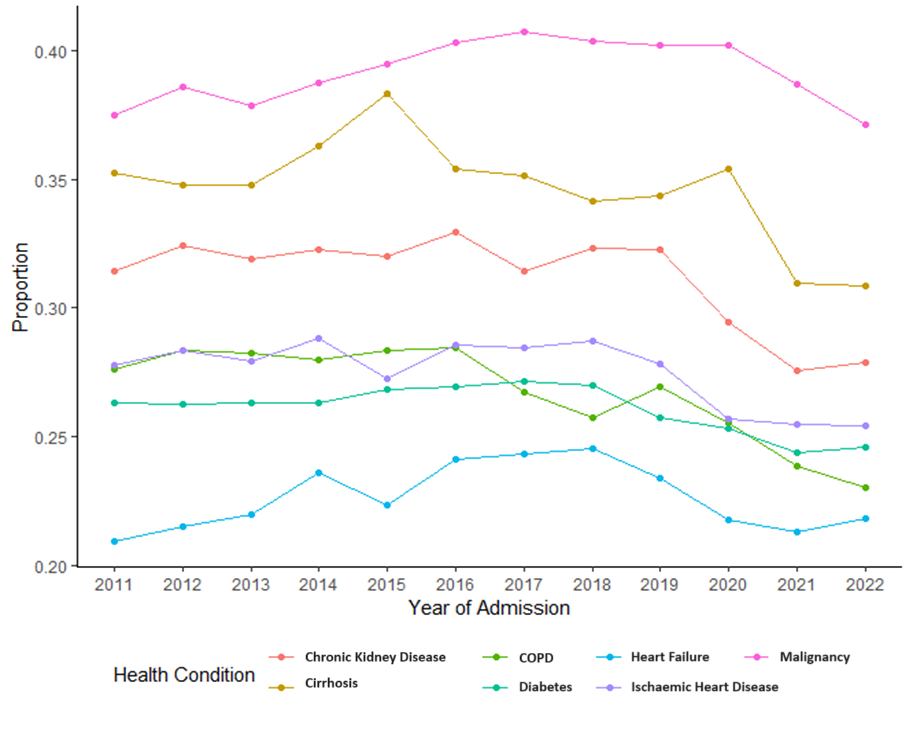
**

**Fig. S6: Proportion of patients with repeat presentations per year**

**
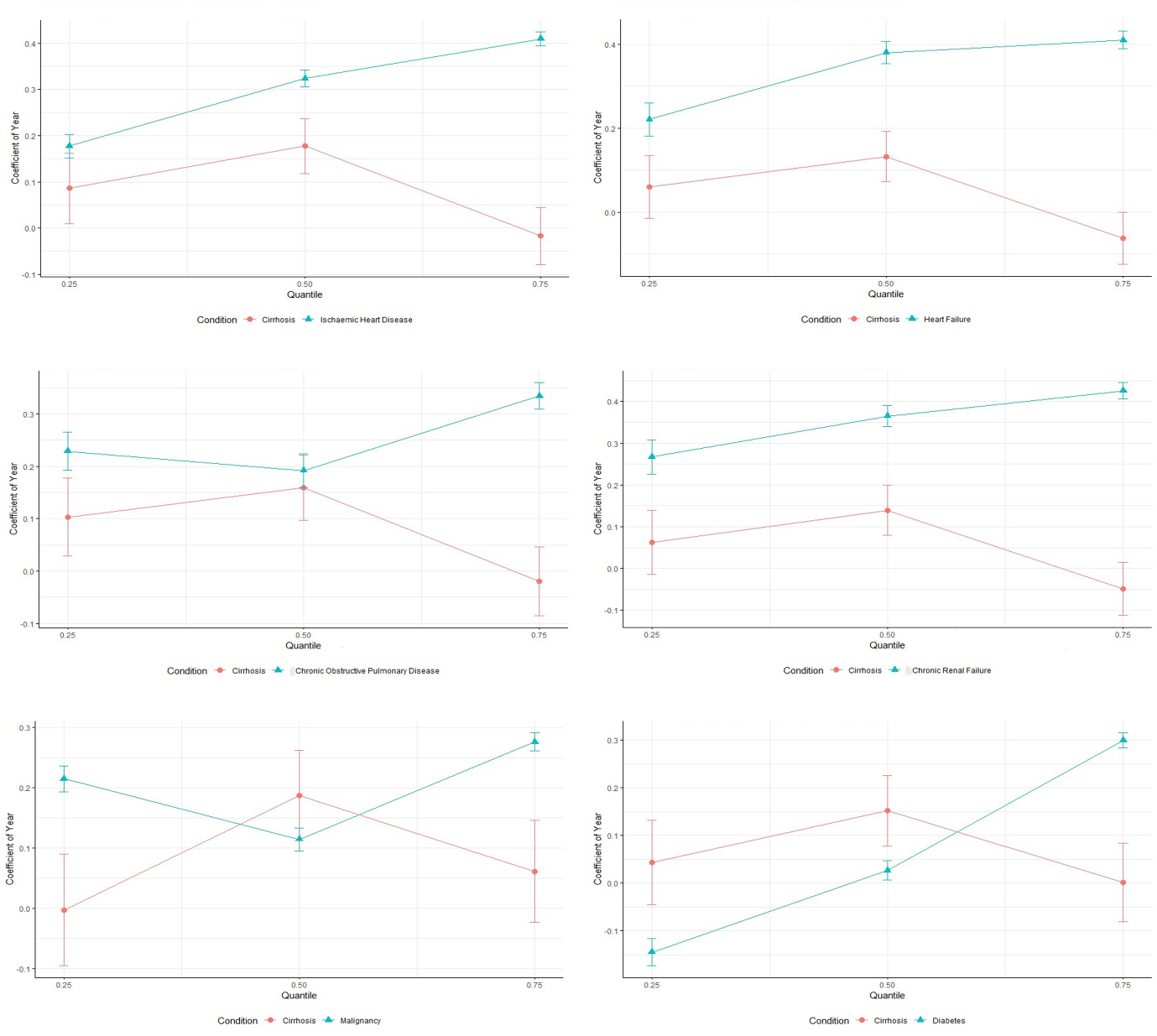
**

**Fig. S7: Quantile regression for change in age at admission per year for various chronic health conditions – Cirrhosis compared to other health conditions**(admissions with both cirrhosis and the health condition being compared against excluded)


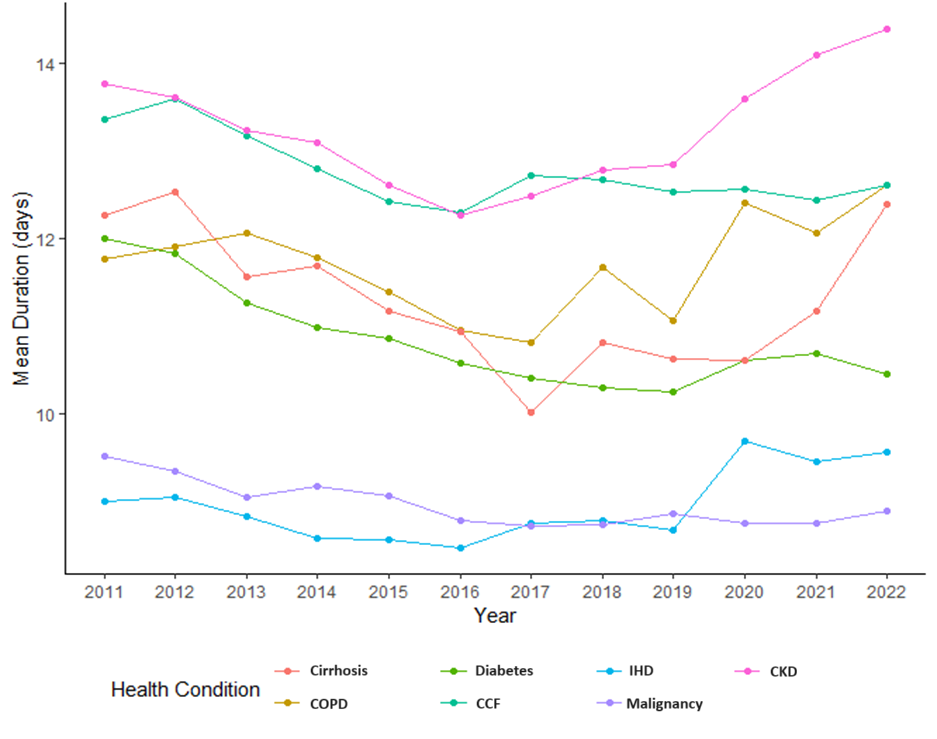


**Fig. S8: Mean duration of hospital admission per year for various chronic health conditions
Abbreviations:** IHD = ischaemic heart disease; CKD = chronic kidney disease; COPD = chronic obstructive pulmonary disease; CCF = congestive cardiac failure

**
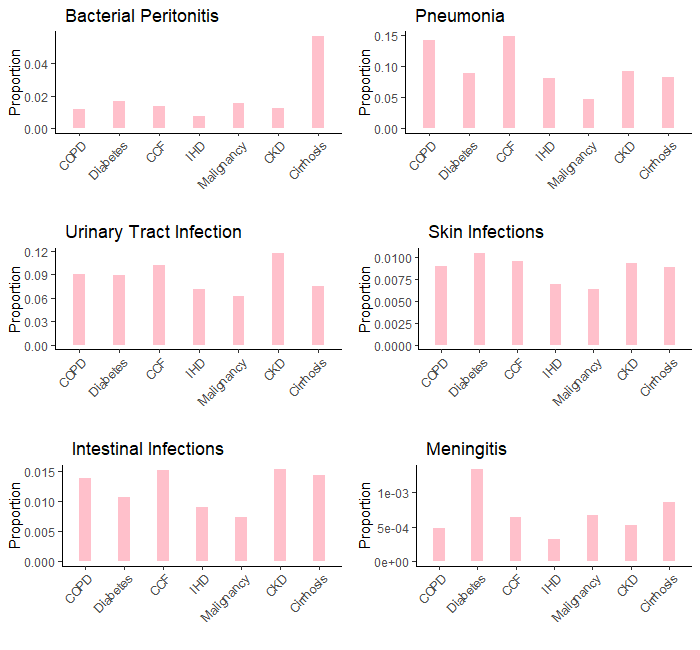
**

**Fig. S9: Proportions of admissions with bacterial infections by presence of particular chronic health conditions
Abbreviations:** COPD = chronic obstructive pulmonary disease; CCF = congestive cardiac failure; IHD = ischaemic heart disease; CKD = chronic kidney disease

**
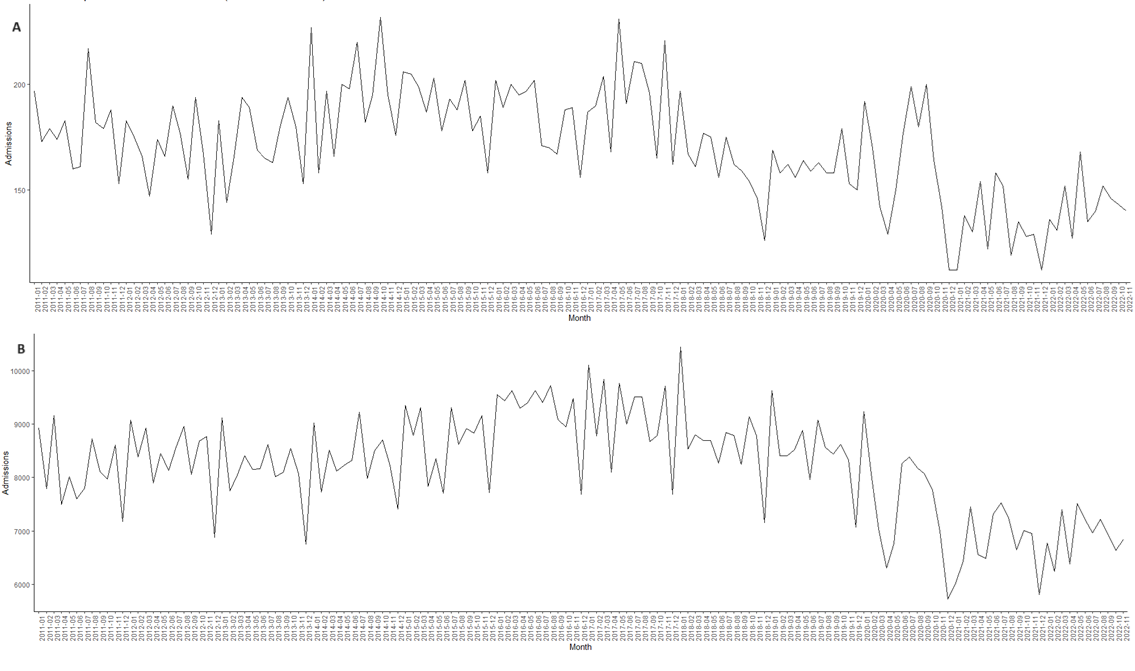
Fig. S10: Admissions per month for A) cirrhosis and B) the combined cohort of all chronic health conditions investigated**

**
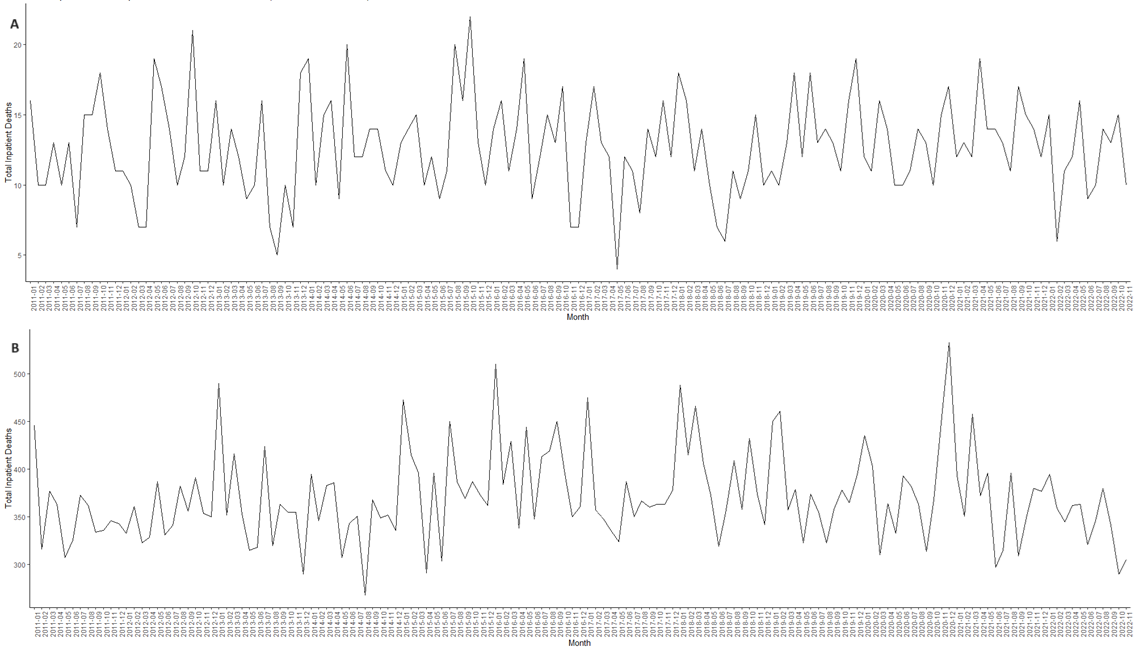
Fig. S11: Inpatient deaths per month for A) Cirrhosis and B) the combined cohort of all chronic health conditions investigated**

**
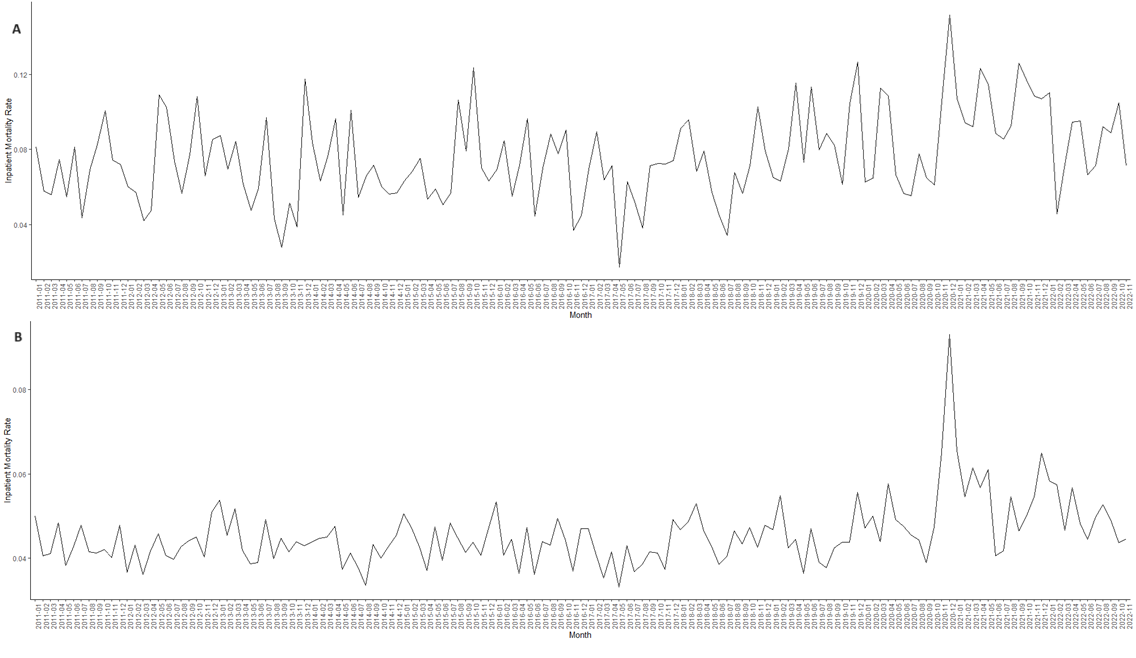
Fig. S12: Inpatient mortality rate per month for A) Cirrhosis and B) the combined cohort of all chronic health conditions investigate**


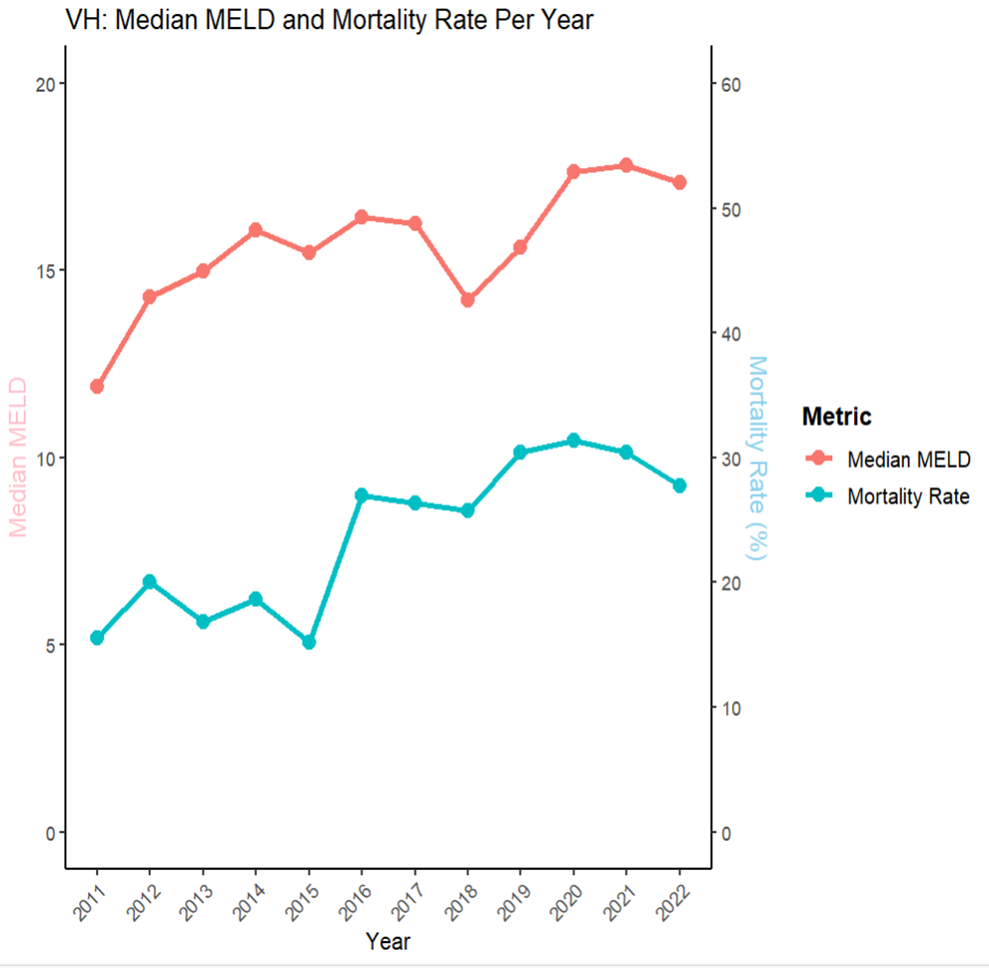


**Fig. S13A: Association Variceal Haemorrhage and in-patient mortality per year. Abbreviations: VH = Variceal Haemorrhage**


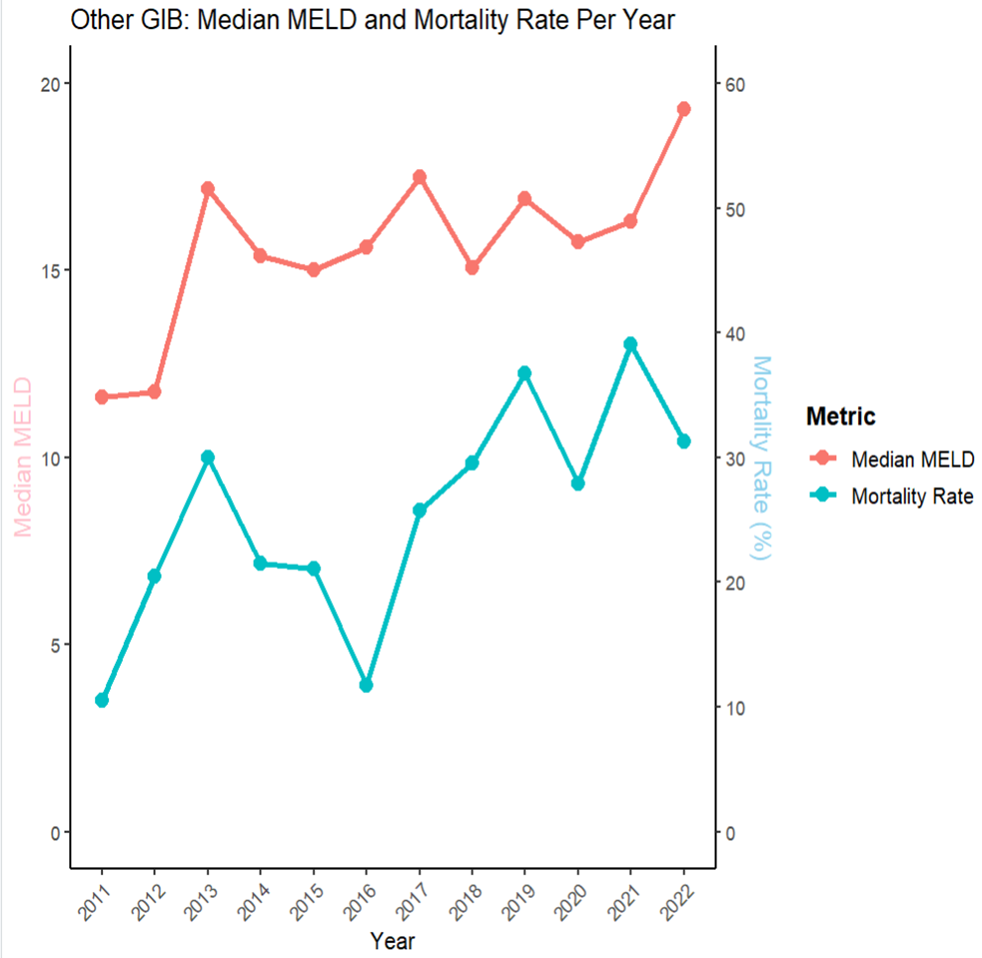


**Fig. S13B: Association between other Gastrointestinal Bleedings and in-patient mortality per year. Abbreviations: GIB = Gastrointestinal Bleeding**


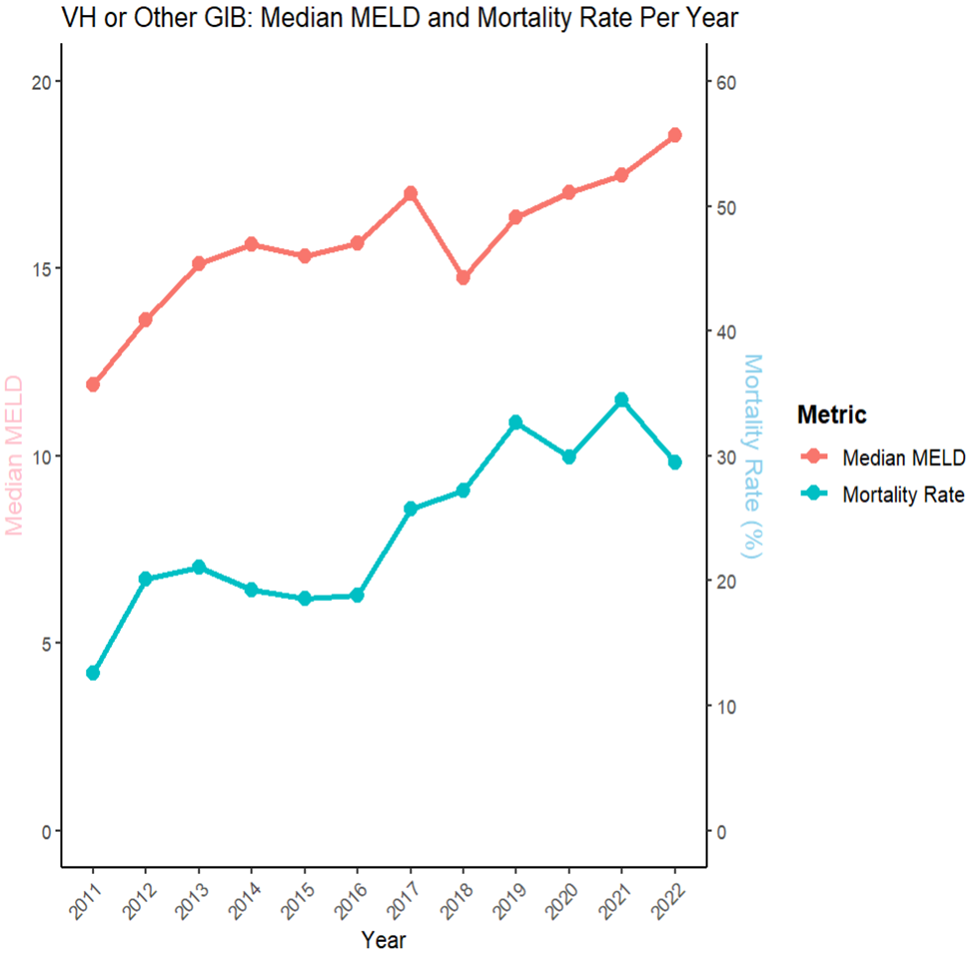


**Fig. S13C: Association between Variceal Haemorrhage and other Gastrointestinal Bleedings and in-patient mortality per year. Abbreviations: VH = Variceal Haemorrhage, GIB = Gastrointestinal Bleeding**
